# Frequency-preference response in covalent modification cycles under substrate sequestration conditions

**DOI:** 10.1038/s41540-021-00192-8

**Published:** 2021-08-17

**Authors:** Juliana Reves Szemere, Horacio G. Rotstein, Alejandra C. Ventura

**Affiliations:** 1 Instituto de Fisiología, Biología Molecular y Neurociencias (IFIBYNE), CONICET-UBA, Buenos Aires, Argentina; 2 Federated Department of Biological Sciences, New Jersey Institute of Technology & Rutgers University, Newark, NJ United States; 3 Departamento de Física, FCEyN UBA, Ciudad Universitaria, Buenos Aires, Argentina

**Keywords:** Signal processing, Computational biology and bioinformatics

## Abstract

Covalent modification cycles (CMCs) are basic units of signaling systems and their properties are well understood. However, their behavior has been mostly characterized in situations where the substrate is in excess over the modifying enzymes. Experimental data on protein abundance suggest that the enzymes and their target proteins are present in comparable concentrations, leading to substrate sequestration by the enzymes. In this enzyme-in-excess regime, CMCs have been shown to exhibit signal termination, the ability of the product to return to a stationary value lower than its peak in response to constant stimulation, while this stimulation is still active, with possible implications for the ability of systems to adapt to environmental inputs. We characterize the conditions leading to signal termination in CMCs in the enzyme-in-excess regime. We also demonstrate that this behavior leads to a preferred frequency response (band-pass filters) when the cycle is subjected to periodic stimulation, whereas the literature reports that CMCs investigated so far behave as low-pass filters. We characterize the relationship between signal termination and the preferred frequency response to periodic inputs and we explore the dynamic mechanism underlying these phenomena. Finally, we describe how the behavior of CMCs is reflected in similar types of responses in the cascades of which they are part. Evidence of protein abundance in vivo shows that enzymes and substrates are present in comparable concentrations, thus suggesting that signal termination and frequency-preference response to periodic inputs are also important dynamic features of cell signaling systems, which have been overlooked.

## Introduction

Biological systems must respond to internal and external variations such as the depletion of nutrients, the fluctuations in hormone levels, and the arrival of sensory signals. In response to stimuli, the pathway-controlling enzymes change their activities. Two basic phenomena play a significant role in this processing: allosteric changes in protein conformation and covalent modification of proteins^1^.

Covalent modification cycles (CMCs) are one of the major intracellular signaling mechanisms, both in prokaryotic and eukaryotic organisms^2^. In such cycles, a signaling protein is modified by the addition of a chemical group. This modification may result in either activation or inactivation, depending on the particular signaling pathway involved, followed by a reverse process, thus closing the cycle. For phosphorylation–dephosphorylation cycles, two opposing enzymes are involved: a kinase and a phosphatase. In the absence of external stimulation, the cycle is in a steady-state where the activation and inactivation reactions are dynamically balanced. External stimuli that produce a change in the enzymatic activity shift the activation state of the target protein, creating a departure from a steady state, which can propagate through a signaling cascade. While individual CMCs are simply elements of a large signaling network, understanding their response to inputs is an essential first step in characterizing the response of more-elaborated signaling networks to external stimuli.

There is a large body of literature on CMCs and cascades of CMCs in the substrate-in-excess regime using mathematical modeling tools^1,3–7^. This regime implies that the substrate abundance is in large excess over the modifying enzymes’ abundances. Importantly, it was predicted that these systems can be highly sensitive to changes in stimuli if their catalyzing enzymes are saturated with their target protein substrates^1^ (note that enzyme saturation is a stronger requirement than substrate in excess). This mechanism was termed zero-order ultrasensitivity and has received enormous attention throughout the years.

However, the substrate-in-excess condition cannot be guaranteed in vivo, because endogenous enzyme concentrations are much higher than those used in a typical in vitro assay^8,9^. In cascades, experimental data on protein abundance suggest that both enzymes and their target proteins are present in comparable concentrations. This is the case of the mitogen-activated protein kinase (MAPK) cascade, which was studied using a combination of theoretical and experimental tools^10^. Estimation of the parameters associated with each level of the cascade demonstrated that the amount of protein in both the second and third levels are similar. In general, in a cascade, the protein at one level operates as the enzyme in the next one, suggesting that at least the two levels involved are not within the substrate-in-excess regime. Enzyme-in-excess scenarios (the opposite of substrate-in-excess) were studied recently supported by in vivo levels of kinases and phosphatases frequently exceeding the levels of their corresponding substrates in budding yeast^11^.

Theoretical work focusing on the departure from the substrate-in-excess plus enzyme saturation conditions is scarce^12,13^. In particular, it is not clear how CMCs in an enzyme-in-excess regime and the cascades of which these CMCs are partly responding to external inputs. It was shown that sequestration of the substrate results in a reduction in ultrasensitivity, which changes the dynamics of a CMC and may account for signal termination and a sign-sensitive delay^14^, in which the rise in the signal is delayed but the dropping signal is transduced immediately. Such a delay element provides cells with units that neglect short fluctuations in signals but transduce long signals^15^. In another study, the importance of sequestration-based feedback in signaling cascades was thoroughly analyzed, and a positive feedback mechanism that emerges from sequestration effects was shown to bring about bistability in the cascade^16^. Negative feedback has been shown to emerge from sequestration effects, as was theoretically predicted^7^ and then experimentally validated^17,18^. Furthermore, the input–output curves were classified in terms of the saturation state of the activating and inactivating enzymes, including the ultrasensitive regime^3^. Only two of the mentioned studies have addressed the response properties of CMCs to fluctuating external inputs^3,17^.

In this paper, we investigate the response properties of CMCs in the enzyme-in-excess regime to both step-constant stimulations (inputs abruptly increasing from zero to a value that remains constant in time) and periodic stimulation resulting from sequestration of the substrate protein. The complexity of the transient response patterns of a dynamical system to constant stimulation ranges from a monotonic increase (relatively simple), to overshoot (intermediate) to damped oscillations. They reflect the different effective time scales present in the system, which are uncovered by the input and result from the interplay of the system’s time constants, and may have different functional consequences. Overshoot responses reflect a property of the underlying system referred to as adaptation or signal termination; i.e., their ability to return to a steady-state value lower than the peak response while the stimulation is still active. Requirements for biochemical adaptation were extensively studied^19,20^. Additional scenarios leading to the same type of signal termination responses include receptor internalization and receptor desensitization^21^ and the response to protein variation^22^. To our knowledge, there are no reports of signal termination in the substrate-in-excess regime, whereas, in the enzyme-in-excess regime, signal termination was predicted^14^, but not investigated in detail. The questions arise of whether signal termination is present only in this enzyme-in-excess regime and under what conditions this occurs. The study of relatively simple systems shows that signal termination may arise from negative feedback or incoherent feedback loop mechanisms^19^. Therefore, understanding the mechanisms underlying signal termination will shed light on the broader problem of characterizing the role of negative regulators in cell signaling^23^. Previous work on cascades in the substrate-in-excess regime has shown the presence of damped oscillations in CMC cascades^7^ in response to constant inputs, raising the question of whether these patterns persist in the enzyme-in-excess regime.

The transient dynamics associated with adaptation reflect an effective time scale of the system, which was shown to have implications for their preferred frequency response to periodic inputs^24–27^. In spite of this, the frequency response of CMCs has received much less attention than adaptive behavior. Some studies found that CMCs behave as tunable low-pass filters, filtering out high-frequency fluctuations or noise in signals and environmental cues^3,17,28,29^. A frequency-preference response was reported in CMCs only under a dose-conservation scheme (dose-conservation implies that either by amplitude or duration compensation, the total dose is kept constant when the frequency increases)^30^. However, all these studies are away from the enzyme-in-excess regime and thus, they preclude signal termination in the unforced CMC.

In this paper, we use mathematical modeling and detailed computational simulations to characterize the conditions under which CMCs in the enzyme-in-excess regime exhibit adaptation to constant inputs (signal termination) and preferred frequency responses to periodic inputs, their properties, how these two phenomena are related, and their consequences for the dynamic behavior of the cascades these CMCs are part of. Specifically, we focus on the regimes where enzymes are in similar or higher amounts than the substrates, resulting in a large fraction of the substrate protein sequestrated by the enzymes. Cascades are not simply chains of CMCs, where one CMC feeds the subsequent one, but because of the backward connections between CMCs, cascades are relatively complex networks^7^ and therefore their dynamics cannot be simply inferred from the dynamics of the CMC components.

Our modeling approach involves the so-called mechanistic models fully describing the interactions between enzymes, substrates, and products overall the time scales present in the process. The mathematical descriptions of the functioning of a CMC usually reduce the system’s dimensionality by means of a quasi-steady-state approximation (QSSA)^31^, leading to a one-dimensional system. However, one-dimensional systems do not exhibit signal termination and preferred frequency responses to periodic inputs in realistic conditions (i.e., unless they are imposed to the system), and therefore these approximations are not applicable to the CMCs we study. Subsequent approximations applicable to the substrate-in-excess regime (Goldbeter & Koshland, 1981) or the so-called total QSSA (tQSSA), which is applicable also when the concentrations of substrate and enzyme are comparable or the enzyme-in-excess regimes (Tzafriri, 2003), also lead to one-dimensional systems and therefore they are also not applicable to our study either. The main reason for these failures is that the fast time scale, which is neglected in the above-mentioned dimensionality process, plays a significant role in producing the two phenomena that are the object of our study. To validate these ideas, we compare our results using the “full” models with the results using approximated models.

Understanding how a response is triggered is as important as deciphering why it terminates and how it can be reactivated. Different time scales emerge from these processes, the response time, the duration of the signal, the recovery time. If the stimulating signal has an associated natural time scale as well, as is the case for time-varying signals, it is expected that interesting behavior could emerge from the interaction of all the involved time scales. Given that in vivo data suggest that sequestration of the substrate is at play in CMCs and in signaling pathways where those CMCs are involved, and given that those pathways are subjected to time-varying signals, understanding their frequency response in sequestration conditions is not only interesting but also relevant.

To summarize, the main focus of the paper is on frequency-preference response in CMCs under substrate sequestration conditions. The paper deals with two system-level behaviors that are not well characterized in CMCs: signal termination and frequency-preference response. It also deals with the enzyme-in-excess regime, a regime that, while physiological, was not properly studied in the literature. The paper studies the connection between having a transient response under constant stimulation, referred to as signal termination, and exhibiting a frequency-preference response under periodic stimulation. Finally, the article also evaluates how the modeling approximations that reduce systems’ dimensionality fail in describing the temporal properties of the responses. These last two features (connection transient response—frequency-preference response, and failure of some simplifying approximations) can be extrapolated to other systems and are not exclusive to CMCs.

The organization of the paper is as follows. We first study CMCs with a mechanistic mathematical model and under substrate sequestration conditions, characterizing signal termination in the parameter space. We then apply periodic stimulation to CMCs exhibiting signal termination, finding conditions for frequency-preference emergence. We then study cascades of CMCs. Finally, we evaluate the performance of the approximations usually employed to describe CMCs, in detecting both signal termination and frequency preferences. We conclude by discussing the potential implications of the results in the article.

## Results

### Signal termination in CMCs under sequestration conditions

**Fig. 1:**
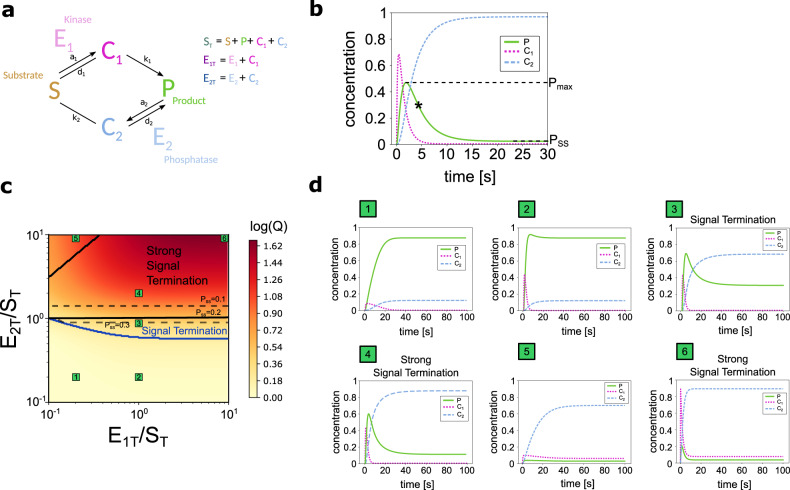
Signal termination in a CMC: the importance of the relative concentrations between substrate and enzymes. **a** Scheme representing a CMC cycle. *S* is the unmodified substrate, *P* the product (modified substrate), *E*_*1*_ and *E*_*2*_ are the modifying enzymes (kinase and phosphatase), *C*_*1*_ is the substrate–kinase complex, *C*_*2*_ is the product-phosphatase complex. Three kinetic parameters are associated with each enzymatic reaction, *a*_*i*_, *d*_*i*_, and *k*_*i*_: the association, dissociation, and catalytic rates, respectively (more details in the Methods section). **b** Phosphorylated protein (*P*, solid green line), substrate–kinase complex (*C*_*1*_, dotted pink line), and product-phosphatase complex (*C*_*2*_, dashed blue line) are plotted versus time. Parameter values: *a*_*1*_ = 3.5; *d*_*1*_ = 1; *k*_*1*_ = 50; *a*_*2*_ = 0.3; *d*_*2*_ = 0.25; *k*_*2*_ = 0.25; *E*_*1T*_ = 100; *E*_*2T*_ = 100; *S*_*T*_ = 35. *a*_*i*_, *i* = 1,2, are in units of 1/(time × concentration), *d*_*i*_ and *k*_*i*_ are in units of 1/time, the total enzymes (*E*_*1T*_ and *E*_*2T*_) are in units of concentration (see Methods section). *P*_max_ and *P*_ss_, the maximum and the steady-state levels of *P*, are indicated over the plot. The characteristic value 0.63 × *P*_max_ is indicated with an asterisk over the time course. **c** Graphs of *Q* (=*P*_max_/*P*_ss_) versus *E*_*1T*_/*S*_*T*_ and *E*_*2T*_/*S*_*T*_, with *Q* in color scale. *E*_*1T*_/*S*_*T*_, *E*_*2T*_/*S*_*T*_, and *Q* are in logarithmic scale, *E*_*1T*_/*S*_*T*_ and *E*_*2T*_/*S*_*T*_ vary between 0.1 and 10, resulting in *Q* values in the range between 1 and ~46. The solid blue line over the plots corresponds to *Q* = 1.6 and separates signal termination from monotonic behavior and non-monotonic behavior not satisfying the criteria. The solid black line over the plot corresponds to *P*_ss_ = 0.2. The cases over the line (*P*_ss_ > 0.2) are termed strong signal termination. Dashed black lines correspond to *P*_ss_ = 0.1 or 0.3, indicating different boundaries for the strong signal termination region. On the upper left triangle, *Q* < 1.6 (no signal termination satisfying the criteria). The kinetic parameter values are as in **b**. **d** Representative time courses in different regions in **c**. Parameter set 1: *E*_*1T*_/*S*_*T*_ = 0.13, *E*_*2T*_/*S*_*T*_ = 0.13, parameter set 2: *E*_*1T*_/*S*_*T*_ = 1, *E*_*2T*_/*S*_*T*_ = 0_._13, parameter set 3: *E*_*1T*_/*S*_*T*_ = 1, *E*_*2T*_/*S*_*T*_ = 0_._79, parameter set 4: *E*_*1T*_/*S*_*T*_ =1, *E*_*2T*_/*S*_*T*_ = 1_._26, parameter set 5: *E*_*1T*_/*S*_*T*_ = 0.13, *E*_*2T*_/*S*_*T*_ = 7.9, parameter set 6: *E*_*1T*_/*S*_*T*_ = 7.9, *E*_*2T*_/*S*_*T*_ = 7.9.

To our knowledge, the only report on signal termination in CMCs focused on the interplay of two enzymes, kinase (activating) and phosphatase (inactivating) in 3:1 concentrations relative to the substrate, thus leading to sequestration conditions^14^. The CMC exhibited a transient (overshoot) response under constant stimulation, therefore, terminating the prolonged response signal while the stimulation is still active. This required a fast kinase with low affinity and a slow phosphatase with high affinity. The fast kinase phosphorylates the available target, but the phosphorylated target is subsequently sequestered by the low-activity high-affinity phosphatase. In the steady-state, most of the target substrate is sequestered by phosphatase. In this section, we investigate whether the three conditions that were found to ensure signal termination in CMCs (enzymes in excess over the substrate, kinase faster than phosphatase, and phosphatase with higher affinity than kinase) are necessary conditions and their relative importance. For the remaining of this article, we refer to kinase and phosphatase as the activating and inactivating enzymes, respectively. Our results are general and apply to CMCs of the form presented in Fig. 1a.

In Fig. 1, we illustrate the phenomenon of signal termination in CMCs (Fig. 1a) for a representative set of kinetic parameter values and analyze the importance of the relative concentrations of the substrate and enzymes (results with an alternative parameter set are included in the Supplementary Information, Supplementary Figure 7). The total amount of substrate (*S*_*T*_), kinase (*E*_*1T*_), and phosphatase (*E*_*2T*_) are conserved. *S*_*T*_ is set to zero for times previous to *t* = 0 s and follows a step-like profile for *t* > 0 s. Studies with step-like profiles starting at non-zero levels are included in the Supplementary Information (Supplementary Figure 9). Similar step-like stimulations but in different parameters are considered in the Supplemental Information (Supplementary Figures 1 and 2) and a discussion on why considering *S*_*T*_ as a signal is included in the Discussion. Additional details and the description of the mechanistic mathematical model used here are presented in Methods. Figure 1b shows the time course of *P*, *C*_*1*_, and *C*_*2*_ under similar conditions as in^14^. *C*_*1*_ and *P* increase fast (*C*_*1*_ faster than *P*) and *C*_*2*_ increases on a much slower time scale. The signal (*P*) terminates (reaches its maximum *P*_max_ and then relaxes to its steady-state value *P*_ss_), whereas the stimulation is still active, because of the sequestration of *P* by *C*_*2*_.

In what follows, it is reasonable to focus on signals that exhibit significant termination according to some criteria, which we define here. First, we normalize the time courses of each variable by their conserved total amount, so that each variable is within 0 and 1, as in Fig. 1b. We neglect those outputs for which *P*_max_ < 0.1. Second, we select those outputs for which *P*_ss_ < 0.63 × *P*_max_. Finally, we require *P*_ss_ < 0.2. This ensures a strong enough signal with a large enough decay to steady-state, which in turn ensures a clear signal peak, and a return to values of *P* close to the pre-stimulation values. We refer to the signals satisfying these three conditions as strong signal termination. For future use, we define the peak-to-steady state amplitude *Q* = *P*_max_/*P*_ss_. The second condition implies *Q* > 1.5873 (~1.6). Although the specific numbers chosen (0.1, 0.63, and 0.2) are somehow arbitrary, similar results to the ones presented in this paper are obtained for other choices (Supplementary Section 5).

In Fig. 1c, we evaluate how signal termination depends on the relative concentrations of kinase/substrate (*E*_*1T*_/*S*_*T*_) and phosphate/substrate (*E*_*2T*_/*S*_*T*_). To this end, we scanned *E*_*1T*_ and *E*_*2T*_ within some range (lower, equal, and higher than *S*_*T*_), whereas keeping all other (kinetic) parameters fixed (same values as in Fig. 1b). The blue curve corresponds to *Q* = 1.6 (log(*Q*) = 0.47)) and separates regions for which *Q* > 1.6 (above, satisfying the decay condition) and *Q* < 1.6 (below). Points above this line correspond to signal termination. A subset of these points (in between the two solid black curves) exhibits strong signal termination (signals satisfy the three conditions discussed in the previous paragraph). On the left upper triangle, there is no signal termination (neither strong nor mild) and the outputs are such that *P*_max_ < 0.1. Examples of the signal (*P*) behavior in each region are presented in Fig. 1d.

Figure 1c demonstrates that the occurrence of both signal termination and strong signal termination is primarily controlled by *E*_*2T*_/*S*_*T*_. This means that the phosphatase has to be in (roughly) similar or higher concentrations than the substrate for the two behaviors to occur, while there are weaker restrictions on the relative concentrations of the kinase and substrate. Supplementary Figure 7 in the Supplementary Information shows a similar analysis for a different choice of kinetic parameters.

We next characterize the emergence of signal termination and its dependence on the kinetic parameters of the CMC model. We will focus on the two kinetic conditions mentioned above (kinase faster than phosphatase, phosphatase with higher affinity than the kinase), which are satisfied for the parameter values used in Fig. 1b (the kinase association and dissociation rates are one order of magnitude higher than those for the phosphatase and two orders higher in the case of the catalytic rate).

The **velocity** *V* of an enzymatic reaction is the rate at which the product is formed. For the two reactions in the CMC cycle, these are given by^31^:

1 $$V = \frac{{\rm{d}}{P}}{\rm{dt}} = V_{{\rm{max}},1}\frac{S}{{K_{m,1} + S}},\;V = \frac{{\rm{d}}{S}}{\rm{dt}} = V_{{\rm{max}},2}\frac{P}{{K_{m,2} + P}}$$

The associated parameters *V*_max_ and *K*_*m*_ for each reaction are, respectively, the maximum reaction velocity, defined as the catalytic rate of the enzyme multiplied by its total amount, and the Michaelis constant *K*_*m*_ = (*d*+*k*)/a that indicates the amount of substrate, leading to a velocity *V*_max_/2 (we omit the indices of *a*, *d,* and *k* for clarity). Equation 1 indicates that at small substrate concentrations the rate of the reaction is linear, with an effective rate constant *V*_max_/*K*_*m*_, while at large substrate concentrations the reaction saturates at its maximum rate *V*_max_. For a fixed amount of enzyme *E*_*T*_, the analysis of the role of the velocity of each reaction in the emergence of signal termination reduces to the analysis of *k*/*K*_*m*,_ and *k*.

The **affinity** *Aff* = 1/*K*_*m*_ of an enzyme for the substrate measures the concentration of substrate that must be present to saturate the enzyme. A high (low) value of *Aff* indicates that a small (large) concentration of substrate is needed to saturate the enzyme.

Simple calculations using the parameter values used in Fig. 1b confirm that the kinase is faster than the phosphatase but has a lower affinity than the phosphatase. In fact, *K*_*m1*_ = 14.6 and *K*_*m2*_ = 1.7 (*Aff*_*1*_ ~ 0.07 and *Aff*_*2*_ ~ 0.59) and the two indicators of the reaction’s velocity, *k*_*i*_/*K*_*m,i*_ and *k*_*i*_, result in 3.4 and 50 for the kinase, 0.14 and 0.25 for the phosphatase.

In order to evaluate the impact of relative velocities and relative affinities of kinase and phosphatase in producing signal termination, we performed the following study where the corresponding model parameters for the opposing enzymes (*a*_*1*_ and *a*_*2*_, *d*_*1*_ and *d*_*2*_, *k*_*1*_ and *k*_*2*_) varied along lines in parameter space with slopes *α*_*a*_, *α*_*d*_, and *α*_*k*_, respectively. More specifically, (*a*_*1*_, *d*_*1*_, *k*_*1*_) = *(α*_*a*_ x *a*_*2*_, *α*_*d*_ x *d*_*2*_, *α*_*k*_ x *k*_*2*_) in the range 0.1–10. When the two opposing enzymes have the same kinetics (*α*_*a*_ = 1, *α*_*d*_ = 1, *α*_*k*_ = 1), these kinetics were selected to be those of the phosphatase in Fig. 1b. This phosphatase-based study (kinase relative to the phosphatase) was complemented with an analogous kinase-based study (phosphatase relative to the kinase) using a parameter *β* with the same characteristics of the parameter *α* above, but when the two opposing enzymes have the same kinetics, these kinetics were selected to be those of the kinase in Fig. 1b.

For the phosphatase-based study, starting from that parameter set, we consider four cases: a common control parameter for the three kinetic parameters (case 1) and a control parameter for any one of the kinetic parameters, whereas the other two remain fixed and equal (cases 2, 3, 4). More specifically,

1. *α*_*a*_ = *α*_*d*_ = *α*_*k*_ = *α*, meaning (*a*_*1*_, *d*_*1*_, *k*_*1*_) = *α*(*a*_*2*_, *d*_*2*_, *k*_*2*_),
2. *α*_*d*_ = *α*_*k*_ = 1 and *α*_*a*_ = *α*, meaning *a*_*1*_ = *α a*_*2*_ and(*d*_*1*_, *k*_*1*_) = (*d*_*2*_, *k*_*2*_),
3. *α*_*a*_ = *α*_*k*_ = 1 and *α*_*d*_ = *α*, meaning *d*_*1*_ = *α d*_*2*_ and (*a*_*1*_, *k*_*1*_) = (*a*_*2*_, *k*_*2*_),
4. *α*_*a*_ = *α*_*d*_ = 1 and *α*_*k*_ = *α*, meaning *k*_*1*_ = *α k*_*2*_ and (*a*_*1*_, *d*_*1*_) = (*a*_*2*_, *d*_*2*_).

The description of the four cases considered for the kinase-based study is analogous.

For all these cases, we derive analytically the effect of the control parameters (*α* and *β*) over the relative effective first-order rate constants *V*_*1*_/*V*_*2*_ and the relative affinities *Aff*_*2*_/*Aff*_*1*_, accordingly, where *V*_*1*_ = *k*_*1*_/*K*_*m,1*_ and *V*_*2*_ = *k*_*2*_/*K*_*m,2*_ (see Supplementary Section 2). A simultaneous increase in *V*_*1*_/*V*_*2*_ and *Aff*_*2*_/*Aff*_*1*_ with increasing values of *α* favors signal termination, so it is expected that *Q* increases with *α*. The opposite effects on *V*_*1*_/*V*_*2*_ and *Aff*_*2*_/*Aff*_*1*_ act against signal termination, so it is expected that *Q* decreases with *α*. Any other situation produces competing effects, so the behavior of *Q* is not straightforward, this behavior indicates which effect has stronger control in signal termination. The resulting *Q* also depends on the reference parameter set.

**Fig. 2:**
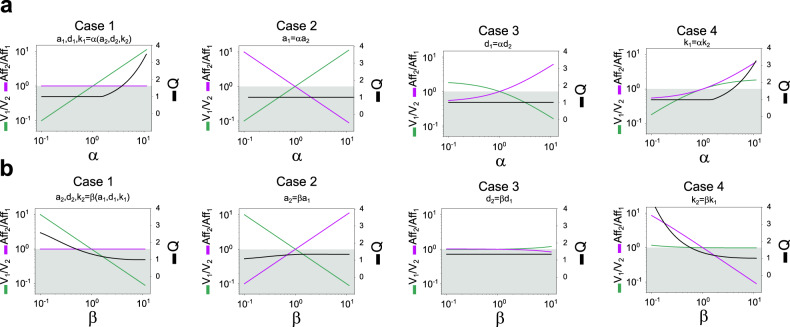
Signal termination in a CMC: kinase faster than phosphatase, phosphatase with higher affinity. **a** Velocity of the kinase (*E*_*1*_) relative to that of the phosphatase (*E*_*2*_) and affinity of the phosphatase relative to that of the kinase (phosphatase parameters values as in Fig. 1b). In each plot, *V*_*1*_/*V*_*2*_ and *Aff*_*2*_/*Aff*_*1*_ are indicated in the left axis while *Q* (=*P*_max_/*P*_ss_) is indicated in the right axis. *V*_*1*_/*V*_*2*_ is plotted in green, *Aff*_*2*_/*Aff*_*1*_ in purple, and *Q* in black. The shadowed region over the plot indicates values of *Q* < 1.6 (no signal termination). **b** Velocity and affinity of the phosphatase relative to those of the kinase (kinase parameters as in Fig. 1b). Other details are as in **a**. Parameter values: *a*_*1*_ = 3.5; *d*_*1*_ = 1; *k*_*1*_ = 50; *a*_*2*_ = 0.3; *d*_*2*_ = 0.25; *k*_*2*_ = 0.25; *E*_*1T*_ = 100; *E*_*2T*_ = 100; *S*_*T*_ = 35.

Our results are presented in Fig. 2. The top panels correspond to the phosphatase-based study and the bottom panels correspond to the kinase-based study. The shadowed region corresponds to *Q* < 1.6 (no signal termination as discussed above). The separating line coincides with *V*_*1*_=*V*_*2*_ and *Aff*_*1*_=*Aff*_*2*_. In all cases, we describe changes as either *α* or *β* increase.

We first analyze the phosphatase-based cases (top panels). In Case 1 (Fig. 2a, Case 1), *V*_*1*_/*V*_*2*_ increases, whereas *Aff*_*2*_/*Aff*_*1*_ remains constant, resulting in *Q* increasing with *α* for *α* higher than 1. Changes along the *a*_*1*_–*a*_*2*_ line (Case 2, Fig. 2a) result in an increase in *V*_*1*_/*V*_*2*_ but a decrease of *Aff*_*2*_/*Aff*_*1*_, leading to competing effects: the kinase increases its velocity but also its affinity with *α*. The resulting *Q* is 1 for all values of *α*, indicating that in this scenario the responses are monotonically increasing. Changes along the *d*_*1*_–*d*_*2*_ line (Case 3, Fig. 2a) result in a decrease in *V*_*1*_/*V*_*2*_ and an increase in *Aff*_*2*_/*Aff*_*1*_, leading to competing effects: the kinase decreases its velocity and its affinity with *α*. As in Case 2, the resulting *Q* is 1 for all values of *α*. Finally, changes along with the *k*_*1*_–*k*_*2*_ line (Case 4, Fig. 2a) result in an increase in both *V*_*1*_/*V*_*2*_ and *Aff*_*2*_/*Aff*_*1*_. Both effects favor signal termination, resulting in an increase in *Q* versus *α*.

A similar analysis for the kinase-based cases shows that in Cases 1 and 2 (Fig. 2b) *V*_*1*_/*V*_*2*_ decreases, whereas *Aff*_*2*_/*Aff*_*1*_ is constant in Case 1 and increasing in Case 2. Case 1 results in signal termination for *β* < 0.5, whereas Case 2 does not present signal termination because the kinase is faster than the phosphatase for *β* < 1, whereas the phosphatase has a higher affinity for *β* > 1. Case 3 (Fig. 2b) exhibits constant values of *V*_*1*_/*V*_*2*_ and *Aff*_*2*_/*Aff*_*1*_, resulting in values of *Q* that do not exceed the threshold for signal termination. Case 4 (Fig. 2b) exhibits almost constant *V*_*1*_/*V*_*2*_ too, but in this case, *Aff*_*2*_/*Aff*_*1*_ is a decreasing function of *β*, resulting in signal termination for *β* < 0.6.

Summarizing the results in Fig. 2, for the two studies, the only two cases resulting in signal termination are Cases 1 and 4 (Fig. 2a, b). Case 4 satisfies the two requirements, *V*_*1*_/*V*_*2*_ > 1 and *Aff*_*2*_/*Aff*_*1*_ > 1, for *α* > 1 or for *β* < 1, so it was expected to achieve signal termination. In Case 1, in contrast, kinase and phosphate have the same affinity, but still results in signal termination when *α* or β are such that *V*_*1*_/*V*_*2*_ > 1. Cases with competing effects in the relations *V*_*1*_/*V*_*2*_ and *Aff*_*2*_/*Aff*_*1*_ resulted in no signal termination. All the calculations related to Fig. 2 are included in Supplementary Section 2.

We now extend the analysis discussed above, which is a local analysis around a selected parameter set (that of Fig. 1b) with the goal of extracting more general conclusions. To this end, we performed a random parameter space exploration in the following ranges: *E*_*1T*_, *E*_*2T*_, and *S*_*T*_ in 10–100, the association and dissociation rates (*a*_*1*_, *d*_*1*_, *a*_*2*_, *d*_*2*_) in 0.1–10, and the catalytic rates (*k*_*1*_, *k*_*2*_) in 1–50. These parameter ranges were determined after a numerical study of the system and based on the literature, as detailed in the Methods section. Modified ranges are presented in Supplementary Figure 8 and do not affect the conclusions of this section. For each simulation, we randomly selected the parameter values within these ranges following the description in Methods, using a Latin Hypercube Sampling. We classified the outputs according to whether they produce signal termination or not. Previous work, mentioned above, provided the following conditions for signal termination^14^: *E*_*1T*_/*S*_*T*_ > 1, *E*_*2T*_/*S*_*T*_ > 1, *V*_*1*_/*V*_*2*_ > 1, *Aff*_*2*_/*Aff*_*1*_ > 1. Therefore, we also classified the output according to whether these conditions are satisfied or not.

**Fig. 3:**
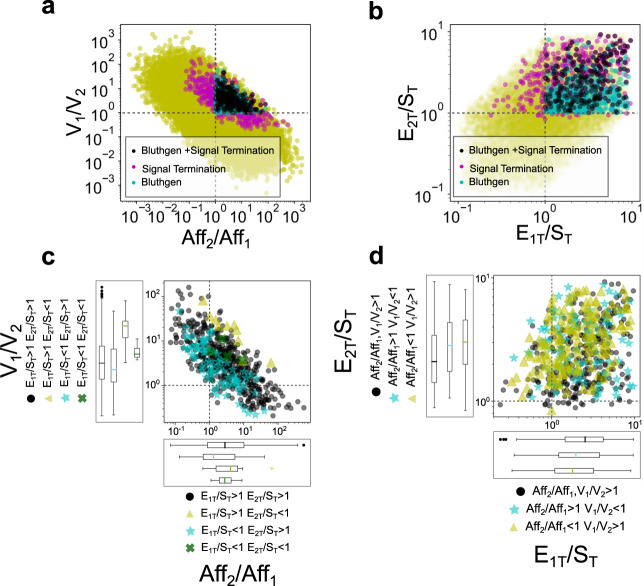
Parameter space exploration. **a***V*_*1*_/*V*_*2*_–*Aff*_*2*_/*Aff*_*1*_ parameter space, **b** *E*_*2T*_/*S*_*T*_–*E*_*1T*_/*S*_*T*_ parameter space. Both panels include the output of numerical simulations for 20,000 parameter sets in the selected ranges of variation. Each dot represents a single simulation output. Dots in purple correspond to outputs with signal termination, dots in cyan correspond to the parameter sets that satisfy Bluthgen’s conditions (see text), dots in black correspond to the parameter sets that satisfy Bluthgen’s conditions and signal termination. All the other simulation results are in yellow. Outputs lower than 0.1 were excluded from the analysis. Since *E*_*1T*_ and *E*_*2T*_ are not kept constant (as was the case in Fig. 2), *V*_*i*_ = *k*_*i*_*E*_*1T*_/*K*_*mi*_ with *i* = 1,2. **c** *V*_*1*_/*V*_2_–*Aff*_*2*_/*Aff*_*1*_ parameter space, but plotting only outputs with signal termination and magnifying the signal termination region. Each output is color and symbol coded according to the value of *E*_*2T*_/*S*_*T*_ and *E*_*1T*_/*S*_*T*_, gray dots if *E*_*2T*_/*S*_*T*_ and *E*_*1T*_/*S*_*T*_ > 1, cyan stars if only *E*_*2T*_/*S*_*T*_ > 1, yellow triangles if only *E*_*1T*_/*S*_*T*_ > 1, green crosses if *E*_*2T*_/*S*_*T*_ and *E*_*1T*_/*S*_*T*_ < 1. **d** *E*_*2T*_/*S*_*T*_−*E*_*1T*/_*S*_*T*_ parameter space, but plotting only outputs with signal termination and magnifying the signal termination region. Each output is color and symbol coded according to the value of *V*_*1*_/*V*_*2*_ and *Aff*_*2*_/*Aff*_*1*_, gray dots if *V*_*1*_/*V*_*2*_ and *Aff*_*2*/_*Aff*_*1*_ > 1, cyan stars if only *V*_*1*_/*V*_*2*_ > 1, yellow triangles if only *Aff*_*2*_/*Aff*_*1*_, green crosses if *V*_*1*_/*V*_*2*_ and *Aff*_*2*_/*Aff*_*1*_ < 1. Horizontal and vertical black lines over the plots indicate *V*_*1*_ = *V*_*2*_, *Aff*_*2*_ = *Aff*_*1*_, *E*_*1T*_ = *S*_*T*_, *E*_*2T*_ = *S*_*T*_, respectively. The characteristics of each distribution are captured in box plots giving the median (color line as central value), the 95% confidence interval of the median, the first and third quartiles (box), the 5th, and 95th percentiles (end of whiskers). Extra symbols in line with the box plots are outliers.

Our results are presented in Fig. 3. In Fig. 3a, b, the yellow dots note all the points in parameter space for which simulations were performed (20,000). The cyan dots note the points in parameter space for which Bluthgen’s conditions are satisfied (696, 3,48%). By construction, the “Bluthgen dots” are located in the first quadrant where two of the four conditions (*E*_*1T*_/*S*_*T*_ > 1, *E*_*2T*_/*S*_*T*_ > 1, *V*_*1*_/*V*_*2*_ > 1, *Aff*_*2*_/*Aff*_*1*_ > 1) are evidently satisfied. If the other two are satisfied as well, these dots in the first quadrant are colored cyan. The purple dots note the points in parameter space for which the output shows signal termination (according to the criteria discussed above) (561, 2.80% from which 442, 2.21%, show strong signal termination). Finally, black dots indicate the overlap between cyan and purple dots, i.e., parameter sets satisfying Bluthgen’s conditions and signal termination (254, 1.27%). The existence of purple not overlapping cyan dots indicates that Bluthgen’s conditions can be relaxed and still obtain signal termination. These conditions cannot be fully violated without losing the signal termination phenomena, as indicated by the fact that no signal termination points lie on the third quadrant of Fig. 3a, and only a few in Fig. 3b. Instead, the relaxation can occur either by extending the conditions to the second quadrant where *Aff*_*2*_/*Aff*_*1*_ < 1 or *E*_*1T*_/*S*_*T*_ < 1, or to the fourth quadrant where *V*_*1*_/*V*_*2*_ < 1 or *E*_*2T*_/*S*_*T*_ < 1. Only a few points exhibit signal termination with *E*_*2T*_/*S*_*T*_ < 1 showing that it is possible, but not robust. Figure 3b also shows that signal termination is more sensitive to condition *E*_*2T*_/*S*_*T*_ > 1 than to condition *E*_*1T*_/*S*_*T*_ > 1: a significantly higher number of signal termination cases lie in the second quadrant than in the fourth one. There are only a few outlier cases not satisfying both conditions (only 4 out of 20,000 within this study).

Figure 3c presents the results of the same simulations, plotting only those cases showing signal termination with a magnification of the quadrants of interest. Figure 3c has a color and symbol code for cases with *E*_*1T*_/*S*_*T*_ and *E*_*2T*_/*S*_*T*_ > 1, only *E*_*2T*_/*S*_*T*_ > 1, only *E*_*1T*_/*S*_*T*_ > 1, none of them. Figure 3d has a color and symbol code for cases with *V*_*1*_/*V*_*2*_ and *Aff*_*2*_/*Aff*_*1*_ > 1, only *Aff*_*2*_/*Aff*_*1*_ > 1, only *V*_*1*_/*V*_*2*_ > 1, none of them. Median values for each group and in each coordinate are indicated with a boxplot on top and on the right side of each panel (details in the Methods section).

A comparative analysis of signal termination and strong signal termination outputs is included in Supplementary Figure 3. From that analysis, we conclude that low values of *V*_*1*_/*V*_*2*_ and high values of *Aff*_*2*_/*Aff*_*1*_ and of *E*_*2T*_/*S*_*T*_ lead to the strong signal termination regime. With *E*_*1T*_/*S*_*T*_ it is not possible to distinguish a region that clearly promotes strong signal termination.

### Frequency-preference response of CMCs to periodic inputs under sequestration conditions

In this section, we study the response of CMCs to time-varying stimuli, in conditions such that they exhibit signal termination under constant stimulation. The presence of adaptation in autonomous linear (and some nonlinear) dynamical systems has been associated with their ability to exhibit resonance, a peak in their amplitude response to oscillatory inputs at a preferred (resonant) frequency. Similar band-pass filtering behavior was reported in other biochemical systems and owing to different mechanisms^32–34^. In the case of CMCs, signal termination and frequency-preference responses result from the complex interaction of effective time scales where the slow time constant of the negative feedback has a prominent role. The question arises whether and under what conditions CMCs that exhibit signal termination also exhibit preferred responses to oscillatory inputs. Although we hypothesized the occurrence of resonant-like responses in CMCs, this is not obvious since the effective time scales leading to signal termination depends on a combination of model parameters (rates) that govern the dynamics of autonomous CMCs.

The impact of the input frequency variation on the response amplitude to oscillatory periodic inputs is typically evaluated by measuring the gain, defined as the output amplitude normalized by the input amplitude as a function of the input frequency. This definition is an extension of the standard impedance for linear circuits, applicable to nonlinear systems under a number of assumptions including (i) the number of input and output cycles coincide, and (ii) the steady-state output amplitude is uniform across cycles when the input oscillations have this property. For simplicity, this type of study is usually conducted by keeping the input amplitude constant for all input frequencies. However, as we discuss below, the presence of conservation laws conditions the ability to use this type of inputs without violating the non-negativity of the substrate (*S*) concentration, and adapted (modified) versions of the input must be used. We note that we are using the word “adaptation” to refer to signal termination in the context of the response to constant inputs and to the modifications needed for the periodic inputs as discussed here. The two are unrelated concepts.

**Fig. 4:**
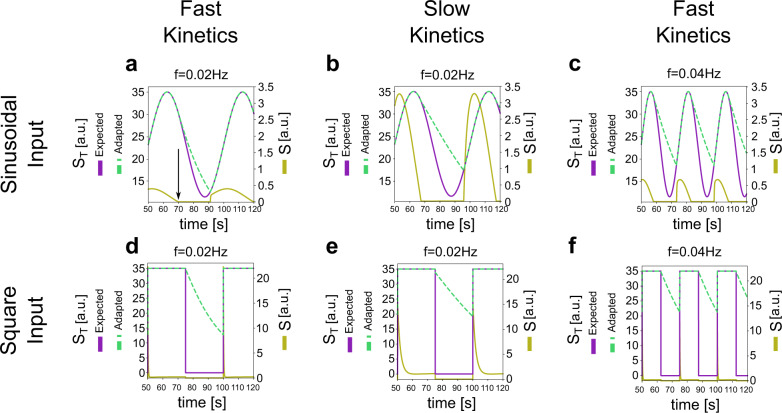
Periodic control of the total amount of substrate. **a**–**c** Sinusoidal variation in *S*_*T*_. The expected variation in *S*_*T*_ (purple solid) is given by *S*_*T*_ = 35(1+0.5sin(*ωt*))/1.5. The accomplished variation in *S*_*T*_ (green dashed) is adapted as explained in the text. The scale on the right of the figure corresponds to *S* (free substrate, solid yellow). The horizontal axis starts at 50 s in order to include stationary variations only. The arrow (a) indicates the time at which complete depletion of *S* occurs (omitted in the other panels). **a** Parameter values: *a*_*1*_ = 3.5; *d*_*1*_ = 1; *k*_*1*_ = 30; *a*_*2*_ = 0.3; *d*_*2*_ = 0.25; *k*_*2*_ = 2.5; *E*_*1T*_ = 100; *E*_*2T*_ = 100; *S*_*T*_ = 35. The frequency of stimulation is *f* = 0.02 Hz, corresponding to *T* = 50 s. **b** Parameter values are as in **a** with the exception of a_1_ and a_2_ that are multiplied by 0.1, resulting in *a*_*1*_ = 0.35 and *a*_*2*_ = 0.03. In this way, the system is overall slower than in **a**. For a slower CMC, the differences between expected and adapted are greater. The frequency of stimulation is *f* = 0.02 Hz. **c** Parameter values as in a but with a frequency of stimulation of *f* = 0.04 Hz, corresponding to *T* = 25 s. The stimulation is faster than in **a**, resulting in greater differences between expected and adapted *S*_*T*_. **d**–**f** Train of square pulses in *S*_*T*_. CMC parameter values as in **a**–**c**.

More specifically, we measure the product (*P*) response to periodic fluctuations in the total substrate (*S*_*T*_). We define the gain as the quotient of the amplitudes of *P* and *S*_*T*_ (see details below). In this section, we aim to apply a periodic control over *S*_*T*_ in a frequency-independent manner by using either sinusoidal and square waves (Fig. 4; expected *S*_*T*_). We refer to this as the expected variation since, as we discuss here, it cannot always be achieved. As indicated in the conservation condition in the scheme of Fig. 1a, the substrate is not always free (*S*), but it is mainly sequestered by the phosphatase (to form the complex *C*_*2*_) and also, but in a lower amount, forming the product (*P*), or forming the complex with the kinase (*C*_*1*_). So, in most scenarios, it is not possible to directly control *S*_*T*_ to follow the expected variation. The closest possible way to do this involves controlling free *S*, either by adding it or washing it out. These changes have an impact on *S*_*T*_ and how it is distributed among the different species. However, when *S* reaches the zero level and the expected variation in *S*_*T*_ indicates that *S*_*T*_ has to decrease even further, the task cannot be achieved instantaneously. Instead, one must cease to add *S* and allow the reactions to progress until a new balance is reached leading to the generation of free *S*, which can be then washed out allowing a further decrease in *S*_*T*_. However, this decrease in *S*_*T*_ due to the recently released *S* being washed out has the time scale given by the reactions, generating a waveform different from that for the expected variation. Furthermore, the minimum value reached by *S*_*T*_ cannot be arbitrarily selected, but it is determined by the process just described. This situation is illustrated in Fig. 4, for both the expected sinusoidal and square wave variation in S_T_ and their adapted versions, which we use in our simulations.

Al alternative approach to adapt the periodic stimulation is evaluated in Supplementary Figure 6 together with an analytical calculation that clarifies the ideas behind the need of adapting the forcing *S*_*T*_ (from expected to adapt) due to the sequestration of some components appearing in the conservation law. This calculation is done in a scenario that is simpler than a CMC, where analytical calculations are not possible (Supplementary Section 4).

**Fig. 5:**
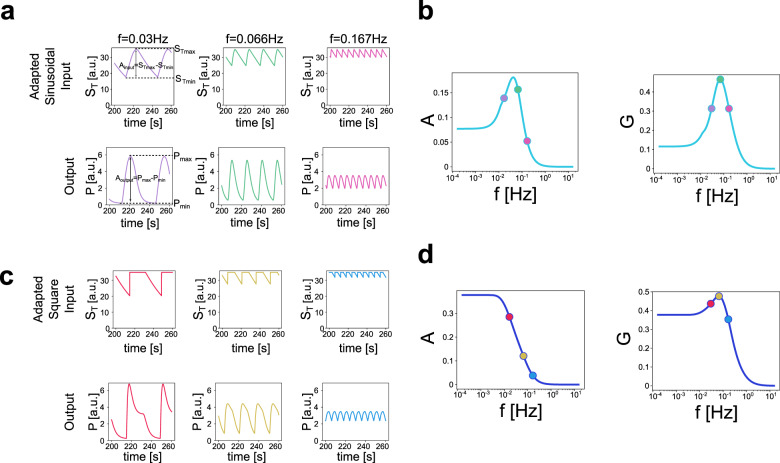
Frequency-preference response in a CMC under periodic stimulation. **a** Representative time courses for adapted sinusoidal inputs *S*_*T*_ (upper panels) with increasing frequencies and the corresponding outputs *P* (lower panels). For the three stimulation frequencies, the maximum reached by *S*_*T*_ is 35, whereas the minimum depends on the frequency, being 17.72, 24.81, and 30.01, in the left, middle, and right panels, respectively. The CMC parameter values are those listed for Fig. 4a and the periods of stimulation are, 33.33, 15.15, 6 s in left, middle, and right panels, respectively, resulting in frequencies of 0.03, 0.066, and 0.167 Hz. **b** Frequency response results, measuring amplitude (left) and gain (right) as a function of the input frequency. The amplitude of the output is defined as the difference between the maximum and the minimum values of *P*. The gain is the ratio between the amplitude of the output and that of the input. The three examples in **a** are indicated over the plot (same color code). **c** Representative time courses for the input *S*_*T*_ (upper panels) and the corresponding outputs *P* (lower panels), corresponding to an adapted train of square pulses stimulation. For the three stimulation frequencies, the maximum reached by *S*_*T*_ is 35, whereas the minimum depends on the frequency, being 20.3, 27.61, and 31.85 in the left, middle and right panels, respectively. Parameter values and input frequencies are as in **a**. **d** Frequency response results, measuring amplitude (left) and gain (right) as a function of the input frequency. The three examples in **c** are indicated over the plot (same color code).

We define the optimal frequency response of the CMC to variations in *S*_*T*_ as the ability of the amplitude or the gain to peak at a non-zero finite frequency (Fig. 5b, d). We measure the amplitudes of *S*_*T*_ and *P* as the difference between the maximum and minimum of these quantities once the output has reached the stationary, periodic variation regime (i.e., disregarding the transients). The amplitude and gain profiles (curves of the output amplitude and gain as a function of the input frequency) are presented in Fig. 5a, b (left: amplitude profiles, right: gain profiles). For both types of input, sinusoidal (Fig. 5a) and square waves (Fig. 5b), the CMC exhibits optimal frequency responses in the gain (Fig. 5b, c, right) respectively, for parameter values for which the underlying autonomous system exhibits signal termination. Figure 5b, d (left) illustrates that the output amplitude does not necessarily capture the optimal response, and this may depend on the type of input used. The abrupt changes in square wave inputs activate the transient overshoots (characteristic of signal termination in the autonomous system) in every input cycle (e.g., Fig. 5c, left), which are more prominent for the lower frequencies (compare Fig. 5b, left and right) and determine the output amplitude. These transients are not explicitly present in the responses to the gradual sinusoidal inputs. Studies with different input amplitudes and with the standard impedance (quotient of the Fourier transforms of the output and the input, in absolute value) are included in Supplementary Figures 4 and 5.

**Fig. 6:**
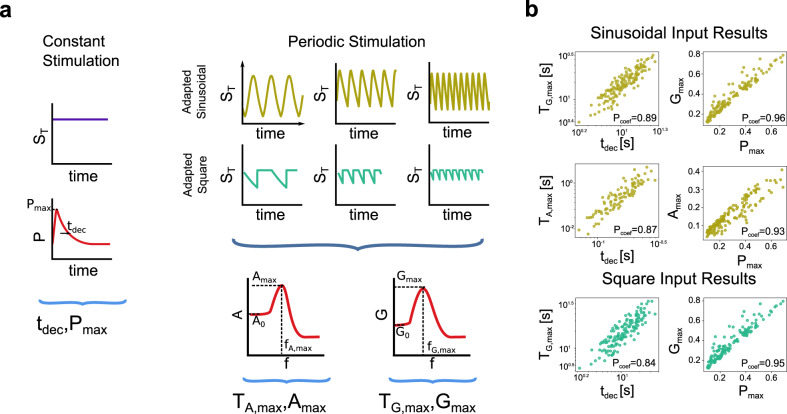
Parameter space exploration for periodic stimulation. **a** Schematic representation of the two different scenarios analyzed, constant (left) and periodic (right) stimulation, and the attributes selected in each one. Constant stimulation is a step in *S*_*T*_ going from zero to the desired value at *t* = 0 and periodic stimulations are the adapted variations, sinusoidal, or train of square pulses, indicated in Figs. 4a–c and 4d–e, with frequencies in the range 10^−4^–10^1^ Hz, resulting in periods in the range 0.1−10^4^ s. For constant stimulation, the attributes are *t*_dec_ and *P*_max_. For periodic stimulations, the attributes are *T*_*A,*__max_, *A*_max_, *T*_*G*__,max_, *G*_max_ (being *T* the period, *T* = 1/*f*). The frequency for which the maximum amplitude/gain is obtained and the corresponding maximum amplitude/gain is indicated over the plots as *f*_*A,*__max_, *f*_*G,*__max_, and *A*_max_, *G*_*ma*x_. **b** Results from the parameter sampling with 5,000 parameter sets. Sinusoidal stimulation was applied as *S*_*T*_(*t*) = *S*_max_(1+0.5sin(2π*ft*))/1.5, then adapted as explained in Fig. 4 (and the text). Train of square pulses stimulation was applied as *S*_*T*_ = *S*_max_ for *nT* < *t* <(*n*+0.5)*T*, *S*_*T*_ = 0 for (*n*+0.5)*T* < *t* <(*n* + 1)*T*, *n* is a natural number, implying the input in the first half of the period. From the 5,000 parameter set values analyzed, 149 resulted in signal termination and all of them exhibited a maximum in the gain versus frequency plot, and also in the amplitude versus frequency plot but for sinusoidal stimulation only. Scatter plots relating an attribute from the output to periodic stimulation and an attribute from the output to step stimulation. The Pearson coefficient for each case is indicated over the plot. Left plots are *T*_*G*__,max_ and *T*_*A,*__max_ versus *t*_dec_, right plots are *G*_max_ and *A*_max_ versus *P*_max_. Scatter plots in dark yellow correspond to sinusoidal stimulation and scatter plots in cyan correspond to square stimulation.

We now turn to study in more detail the relationship between signal termination (in response to step inputs) and the preferred frequency responses of CMCs to oscillatory inputs. For each parameter set that exhibits signal termination (Fig. 3), we computed the output amplitude and gain profiles as described above (Fig. 5). We investigated the relationship between the two phenomena by comparing a number of representative attributes for the corresponding graphs. For the *P* vs *t* graph, we define *t*_dec_ and *P*_max_ (Fig. 6a, Constant Stimulation) as the time it takes *P* to decrease from its maximum to 63% of it and the maximum value of *P*, respectively. For the amplitude and gain profiles, we define (i) *A*_*0*_ and *G*_*0*_ as the amplitude and the gain obtained at the lowest frequency analyzed, (ii) *f*_*A*__,__max_ and *A*_max_ as the frequency and the amplitude at the preferred frequency (if there is one), (iii) *f*_*G*__,max_ and *G*_max_ as the frequency and the gain at the preferred frequency (if there is one), and (iv) *Q*_*A*_ and *Q*_*G*_ as the ratios between the *A*_max_ and *A*_*0*_, and *G*_max_ and *G*_*0*_, respectively. In addition, we use the preferred periods *T*_*A,*__max_ and *T*_*G,*__max_ corresponding to the *f*_*A*__,max_ and *f*_*G*__,max_ (*T*_*A,*__max_ = 1/*f*_*A*__,max_ and *T*_*G,*__max_ = 1/*f*_*G*__,max_). All these attributes are indicated in Fig. 6a and the definitions are included in the Methods section (Table 1).

**Table 1:** List and definitions of attributes analyzed in the article.

***P***_**max**_ maximum value reached by *P* in its time course.
***P***_**ss**_ steady-state value reached by *P* in its time course.
***t***_**dec**_ time it takes *P* to decrease from its maximum value to 63% of it.
***Q*** = *P*_max_/*P*_ss_
***A*** (amplitude) maximum minus minimum values in the time course of variable *P*, normalized by *S*_*T,*max_ (*S*_*T*_ maximum value).
***A***_***0***_ normalized amplitude obtained at the lowest frequency analyzed.
***A***_**max**_ normalized amplitude at the preferred frequency.
***f***_***A,*max**_ preferred frequency in the amplitude versus frequency plot.
***T***_***A,*max**_ preferred period, *T*_*A,*max_ = 1/ *f*_*A*_,_max_
***Q***_***A***_ = *A*_max_/*A*_*0*_
***G*** (gain) ratio between the amplitude of the output *P* versus that of the input *S*_*T*_.
***G***_***0***_ gain obtained at the lowest frequency analyzed.
***G***_**max**_ gain at the preferred frequency.
***f***_***G,*max**_ preferred frequency in the gain versus frequency plot.
***T***_***G,*max**_ preferred period, *T*_*G,*__max_ = 1/ *f*_*G*_,_max_
***Q***_***G***_ = *G*_max_/*G*_*0*_

As for signal termination in Section 1, we define a number of criteria to establish the significance of preferred frequency response and we focus on the significant cases. First, both the amplitude and the gain are in the range 0–1 (the amplitude because of being normalized by *S*_*T,*max_ and the gain because *P* is part of the conservation condition of *S*_*T*_ stated in Fig. 1a, so *P* is always lower than *S*_*T*_). We discard cases with a maximum amplitude/gain lower than 0.1. Second, we select those outputs for which *A*_*0*_ < 0.9 *A*_max_ and *G*_0_ < 0.9 *G*_max_. This ensures a significant gain with a large enough maximum. To characterize the strength of the preferred frequency response, we define the peak-to-initial value *Q*_*A*_ = *A*_max_/*A*_*0*_ in the amplitude profiles, and *Q*_*G*_ = *G*_max_/*G*_*0*_ in the gain profiles. This implies *Q*_*A*_, *Q*_*G*_ > 1.1. While the specific numbers chosen (0.1, 0.9) are somehow arbitrary, similar results to the ones presented in this paper are obtained for other choices. We refer to the parameter sets leading to these two conditions under periodic stimulation, as having frequency preference.

The results of the parameter space exploration indicate that for all the parameter sets for which the CMCs show signal termination, they also show a preferred frequency response in both the amplitude and gain to sinusoidal stimulation. For square wave stimulation, this conclusion holds only for the gain since the amplitude does not show any preferred frequency response. Furthermore, we observed no cases where CMCs exhibit preferred frequency responses without signal termination. In Fig. 6b, we present the correlation graphs between the attributes for signal termination (abscissas) and the preferred frequency responses (ordinates). We include those exhibiting higher correlations, as measured by the Pearson coefficient. The correlation between the decay time and the preferred periods (Fig. 6b, left) indicates that the effective slower time scales of the autonomous CMCs play a significant role in determining their preferred responses to sinusoidal inputs. Unlike linear systems, these time scales are not explicit as model time constants, but involve a combination of model parameters. These results show that input is more effective in producing a significant output if it allows the system to terminate the signal before the new stimulation cycles begin. The correlations between *A*_max_ and *G*_max_ confirm the relationship between the time responses of CMC cycles to constant pulses and the frequency responses to oscillatory inputs. Although these results may not be completely unexpected, they are also not obvious for CMCs, particularly taking into account that CMCs are not explicitly designed as negative feedback loops.

**Fig. 7:**
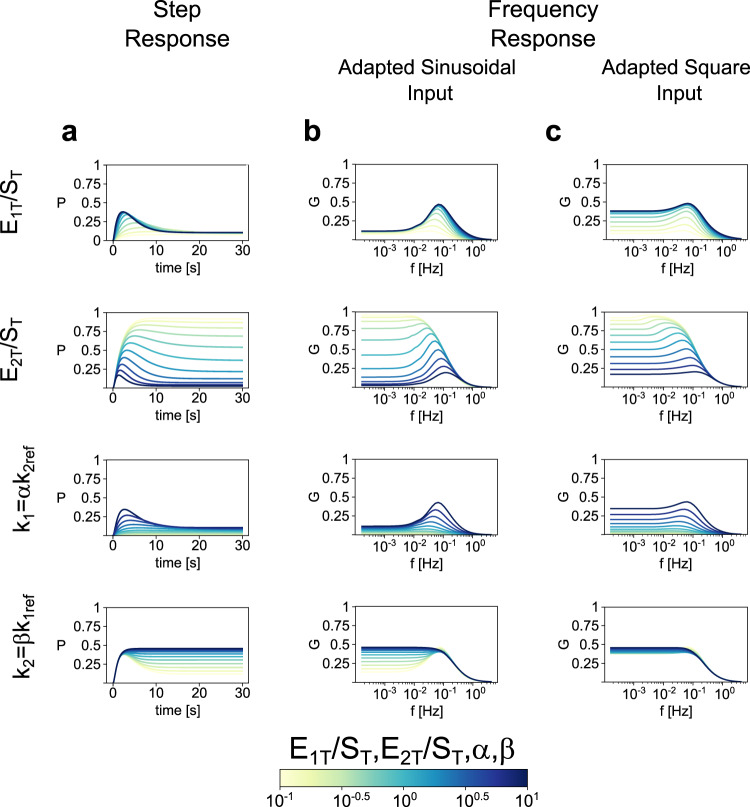
Analysis of the mechanisms leading to frequency preference. **a** P versus time for step (constant) stimulation. **b** Gain versus frequency under sinusoidal stimulation. **c** Gain versus frequency under the train of square pulses stimulation. Each row corresponds to different parameter variations controlling the transition in or out of the signal termination region. First row, *E*_*1T*_/*S*_*T*_; second row, *E*_*2T*_/*S*_*T*_; third row, *k*_*1*_ = *αk*_*2re*f_; fourth row, *k*_*2*_ = *βk*_*1ref*_, with *k*_*2ref*_ and *k*_*1ref*_ the corresponding parameter values for a reference set (as in Fig. 5). These parameter variations are color-coded as indicated in the color bar at the bottom of the figure.

We now turn to the analysis of the biochemical mechanisms of generation of preferred frequency responses in the gain and we compare them with the mechanisms of signal termination to further strengthen the relationship between the two phenomena. These mechanisms consist of the transition from a low-pass filter to a band-pass filter as the parameter under consideration increases. We have identified a number of paths in parameter space that lead to the generation of preferred frequency responses in the gain. They are summarized in Fig. 7. They involve the changes in the quotient of two model parameters (all other parameters fixed) and therefore each of these paths is degenerate in the sense that multiple combinations of model parameters satisfy the same constant constraint for the quotient (Fig. 7b, c).

In principle, from a low-pass filter, preferred frequency responses in the gain profile can be generated as the result of the increase of a parameter value (or combination of parameter values) because (M1) *G* increases faster for intermediate frequencies than for lower frequencies, or (M2) *G* decreases faster for lower frequencies than for intermediate frequencies.

Increasing values of *E*_*1T*_/*S*_*T*_ generate preferred gain responses by mechanism M1 (Fig. 7b, c, row 1) and it is more pronounced and robust for sinusoidal than for square wave inputs (compare panels b and c). For the latter, there is a transition to a low-pass filter as *E*_*1T*_/*S*_*1T*_ continues to increase.

In contrast, increasing values of *E*_*2T*_/*S*_*T*_ generate preferred gain responses by mechanism M2 (Figs. 7b, c, row 2). As for the previous case, the resonances are more pronounced and robust for sinusoidal than for square wave inputs (compare panels b and c), and for the latter, there is a transition to a low-pass filter as *E*_*2T*_/*S*_*2T*_ continues to increase. Therefore, decreasing values of *E*_*2T*_/*S*_*T*_ also generate preferred frequency responses in gain for square wave inputs by mechanism M1.

Increasing values of α generate preferred gain responses by mechanism M1 (Fig. 7b and c, row 3), which are also more pronounced and robust for sinusoidal than for square wave inputs (compare panels b and c) and, for square wave inputs the preferred frequency response is terminated as α continues to increase. Finally, decreasing values of *β* generate preferred gain responses by mechanism M2 (Fig. 7b and c, row 4), which are also more pronounced and robust for sinusoidal than for square wave inputs (compare panels b and c).

### Cascades of CMCs

**Fig. 8:**
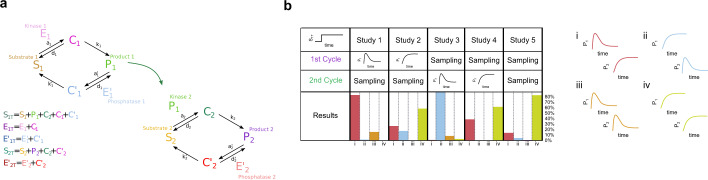
Different regimes in cascades of CMCs. **a** Scheme representing a cascade with two CMC cycles. *S*_*1*_ and *S*_*2*_ are the unmodified substrates, and *P*_*1*_ and *P*_*2*_ are the products (modified substrates), in each level. *E*_*1*_ and *E*_*1*_*’* are the modifying enzymes or kinase and phosphatase in the upper level, *C*_*1*_ and *C*_*1*_*’* are the complexes substrate–kinase and substrate-phosphatase in the upper level, *P*_*1*_ and *E*_*2*_*’* are the modifying enzymes in the lower level, *C*_*2*_ and *C*_*2*_*’* are the complexes in the lower level. The three kinetic parameters associated with each enzymatic reaction are *a*_*i*_, *d*_*i*_, *k*_*i*_, the association, dissociation, and catalytic rates, respectively (more details in the Methods section). The conservation conditions are listed in the scheme. **b** Table summarizing the five representative numerical studies carried out. In all of them, step stimulation is applied. In studies 1 and 2, parameter values of the first cycle are selected so that this cycle in isolation exhibits signal termination or monotonic response, respectively, whereas the parameter values of the second cycle are randomly selected from predefined ranges. In studies 3 and 4, parameter values of the second cycle are selected so that this cycle in isolation exhibits signal termination or monotonic response, respectively, whereas the parameter values of the first cycle are randomly selected from predefined ranges. In Study 5, all the parameter values, first and second cycle, are randomly selected from the mentioned ranges. In all, 5,000 results of numerical simulations were analyzed in studies 1–4, and 10,000 in Study 5. Parameter values for each study are listed in Supplementary Table 1. The ranges for random exploration are concentrations: 10–100, association and dissociation rates: 0.1–10, and catalytic rates: 1–50. The results were classified into four groups (right): (i) those exhibiting signal termination in the first cycle but not in the second one (red bar); (ii) the inverse of (i) (light blue bar); (iii) those exhibiting signal termination in both cycles (orange bar); (iv) those with monotonic behavior in both cycles (light green bar). These groups are schematized next to the table (with the same color pattern as in the last row of the table). The criteria used to classify the outputs in groups are described in Methods. The lowest row in the table contains the results of each study, with a bar plot representing the percentage of cases in groups i–iv.

In this section, we consider a cascade composed of two CMCs, as indicated in Fig. 8a where the first cycle (CMC_1_) is subject to constant or periodic stimulation and we examine how these signals are processed across the network. More specifically, we first study whether signal termination (under constant stimulation) is affected by the presence of a downstream cycle, if it can be propagated downstream in the cascade, and if new behavior emerges from the coupling of cycles. We then characterize the frequency response of the cascade under periodic stimulation. For simplicity, we restrict our study to the gain in response to sinusoidal stimulation and we leave out the details corresponding to the amplitude. We emphasize that a cascade is not simply a feedforward network where CMC_2_ responds to the output from CMC_1_, but it is an interconnected network where CMC_2_ affects CMC_1_ and therefore knowledge from the CMC_1_ output is not enough to predict the CMC_2_ output.

We summarize our studies and results for signal termination in Fig. 8b. In the first two studies (study 1 and study 2), we selected specific parameter values for the first cycle (CMC_1_) and the parameter values for the second cycle (CMC_2_) were randomly distributed. In the following two studies (study 3 and study 4) we selected specific parameter values for the second cycle (CMC_2_) and the parameter values for the first cycle (CMC_1_) were randomly distributed. In study 1 or 3, CMC_1_ or CMC_2_, respectively, exhibits signal termination (under constant stimulation) and preferred frequency response (periodic stimulation). In study 2 or 4, CMC_1_ or CMC_2_, respectively, exhibits no signal termination and therefore no preferred frequency response. In study 5, the parameter values of CMC_1_ and CMC_2_ were randomly selected. There are four possible types of responses for the cascade (Fig. 8b, right). The last row in the table indicates the percentage of each of them. We briefly report our results below and present a comprehensive table comparing the results of the five studies in Supplementary Figure 11).

In study 1, we found that ~83% of the explored parameter sets (initial exploration: 5,000 sets, significant responses: 4,212, see Methods for a detailed description of the criteria) resulted in the persistence of signal termination for CMC_1_, whereas CMC_2_ responds monotonically. A smaller number of cases resulted in signal termination in both cycles (~15%). These numbers indicate that signal termination is eliminated ~2% of the time. Why can the second cycle reduce the signal termination property in the first cycle?. The presence of the second cycle introduces a competitive inhibition effect for the reverse reaction in the first cycle, i.e, the one converting the product back to substrate. This competitive inhibition results in an effective reaction velocity with an increased Michaelis–Menten constant *Km*^31^. Since the phosphatase affinity for the product is the inverse of this *Km*, the presence of the second cycle decreases the phosphatase affinity, whereas the kinase affinity stays unmodified. As indicated in Fig. 3, a high ratio *Aff*_*2*_/*Aff*_*1*_ promotes signal termination, so the competitive inhibition effect that effectively reduces *Aff*_*2*_ results in a decrease in the number of cases with signal termination.

In study 1, most but not all of the cases with signal termination in CMC_1_ resulted in frequency preference when periodically stimulated (65%). This is different from our expectation from the responses of individual CMCs to periodic stimulation (where all cases showing signal termination also showed frequency-preference response). Together, although both signal termination and frequency preference are not always propagated by the cascade, they persist in a non-negligible number of cases.

In study 2, we found that the absence of signal termination in the isolated CMC_1_ does not necessarily cause the cascade to show a monotonic response. In fact, we found signal termination in CMC_1_ only in 26% of the cases. In this case, sequestration of the first cycle substrate in its modified form, i.e., the product, by the substrate of the second cycle reduces the available first cycle substrate for the first cycle kinase and phosphatase, leading to an enzyme-in-excess regime. This situation is not general and depends on the parameter values, resulting in 26% of signal-termination new cases. An alternative explanation is that by adding the second cycle there is an extra term of sequestration of the product of the first cycle, contributing in this way to terminate the signal (see Supplementary Figure 10). Although signal termination in CMC_2_ is not necessarily unexpected taking into account that a monotonic response can rapidly reach steady-state, therefore mimicking an effective constant stimulation situation to CMC_2_, particularly when the isolated CMC_2_ shows signal termination, signal termination in CMC_1_ is a network effect.

In study 3, we found that adding an upstream cycle does not remove signal termination in CMC_2_. Study 4 indicates that is very unlikely that an upstream cycle can lead CMC_2_ into signal termination when isolated produces a monotonic response. In study 5, we also found signal termination and frequency preference in both cycles (signal termination: 14% in CMC_1_, 4% in CMC_2_, 0.7% in both; frequency preference: 5% in CMC_1_, 0.6% in CMC_2_, 0.44% in both).

**Fig. 9:**
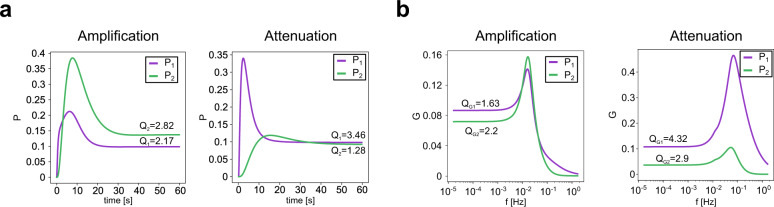
Amplification and attenuation in cascades of CMCs. **a** Representative example of time courses exhibiting amplification (left) or attenuation (right) when stimulated with a step. **b** Representative examples of gain versus frequency plots exhibiting amplification (left) or attenuation (right) when periodically stimulated.

Of particular importance are the network processing capabilities of cascades in controlling the properties of signal termination and frequency-preference responses (to constant and oscillatory inputs, respectively). To illustrate these issues, here we focus on those outputs from study 1 exhibiting signal termination and frequency preference in both cycles of the cascade. In Fig. 9, we present examples of both cascade amplification and attenuation in both signal termination (Fig. 9a) and frequency preference (Fig. 9b). We used the following metrics for each CMC: *Q* = *P*_max_/*P*_ss_ and *Q*_*G*_ = *G*_max_/*G*_*0*._

### Signal termination and frequency-preference response is not captured by usual CMC’s approximations

Modeling studies of enzymatic reactions often rely on a number of simplifying assumptions. Perhaps, the best known is the Michaelis–Menten approximation, which reduces enzymatic reaction models to single differential equations (one-dimensional systems). A key assumption is the identification of a fast time scale governing the evolution of the initial, transient increase in the complex concentration, which then relaxes to zero on a much slower time scale giving rise to the product increase^35,36^. This dimensionality reduction approximation affects minimally the dynamics of the product increase provided a good estimation of the complex concentration after the short transient. For this and other systems where the dynamics are quasi-one-dimensional, the elimination of the transient dynamics is practically inconsequential. In contrast, their response to external inputs fails to capture the response of original systems. Two prototypical examples are signal termination (in response to constant inputs) and the preferred frequency responses to oscillatory inputs. In linear systems, for example, these two phenomena are present in two-dimensional models for the appropriate parameter regimes (e.g., slow negative feedback effect), but they are absent for their quasi-one-dimensional approximation where the autonomous transients are neglected. For signal termination, the explanation is rather simple. For signal termination to occur, the temporal profile of the measured variable achieves a maximum prior to reaching the steady-state (and different from it). For this to happen, the action of a second variable opposing the increase is necessary. Otherwise, the principle of uniqueness for well enough behaved (one-dimensional) systems would be violated, and therefore the steady-state and maximum coincide. For the preferred frequency response, the necessity of higher-than-one dimensionality is predicted by standard calculations. The dynamic explanation is more involved and it derives from the dynamical system analysis carried out^24,37^. These two phenomena are not observed in single enzymatic reactions, as they lack the main ingredients (slow negative feedback), but, as we showed, they are present in networks of enzymatic reactions.

Our study in this paper involves the mechanistic formulation of CMCs (and cascades) without any simplifying assumption. In this section, we examine the effectiveness of the simplifying assumptions typically used for modeling CMCs in reproducing signal termination and frequency preference. We do not intend to carry out a systematic analysis, but rather to provide some insight into the issues and the motivation for our modeling decisions.

**Fig. 10:**
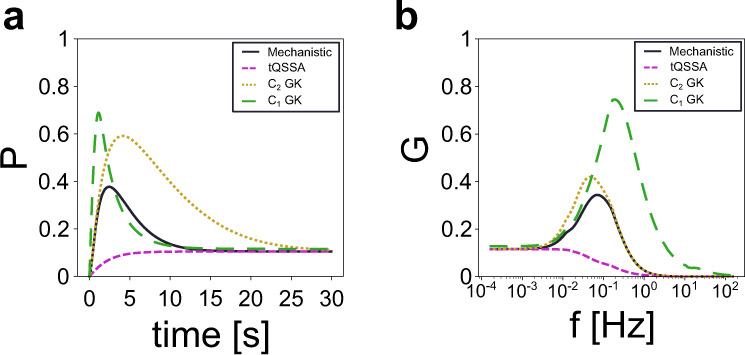
Approximation comparisons. **a** Time courses for variable *P* obtained by using the mechanistic model (black solid), the tQSSA (orange dashed), and a combined mechanistic model with a Goldbeter–Koshland approximation for only one of the modified enzymes (purple and green). **b** Gain versus frequency plots for the same conditions as in **a**. Parameter values as in Fig. 5.

The QSSA is also frequently used to derive reduced models for enzyme-catalyzed reaction networks (as CMCs). Under the additional substrate-in-excess assumption (the substrate is in excess over the enzymes), Goldbeter and Koshland derived a single differential equation for the modified substrate P^1^ (details in the Supplemental Information). The applicability of this last model is restricted to conditions when the substrate concentration is much higher than that of the converter enzymes. In order to correct for the failure of the approximation to capture the transient dynamics, another approximation called the tQSSA was designed^38^. It is based on certain linear combinations of the original variables and it has proven to yield much better approximations, especially when the enzyme concentrations become comparable to or larger than that of the substrate^6^. However, the description of a CMC with a tQSSA also leads to a single differential equation for the modified substrate *P* (details in the Supplemental Section 7), and therefore it is unable to capture both signal termination and the frequency-preference response to oscillatory inputs (Fig. 10).

To examine whether a mixed type of approximation that reduces the model dimensionality, but results in a two-dimensional (rather than one-dimensional) model, is able to capture both signal termination and the frequency preferred responses, we combined the mechanistic model with a Goldbeter–Koshland type of approximation for only one of the enzymes leading to two reduced models (*C*_*1*_ GK and *C*_*2*_ GK), one of each enzyme (*E*_*1*_ and *E*_*2*_, respectively). Our results (Fig. 10, purple and green lines) demonstrate that while these reduced models show signal termination and frequency-preference responses, the resulting patterns are not good approximations for the full, mechanistic model.

## Discussion

CMCs and cascades of CMCs have been studied both theoretically and experimentally as the primary intracellular signaling mechanisms in living systems^1,3,7,17,18,29,39,40^. An important question is how these systems respond to external stimulation. Two prototypical stimulation signals are step-constant (abrupt transitions between zero stimulation and a constant positive stimulation) and periodic. Step-constant stimulation serves the dual purpose of characterizing the variety of steady states available to CMCs and uncovering the transient dynamics leading to these states. In the simplest case, these dynamics are monotonic. However, recent work has shown that CMCs have the ability to exhibit signal termination^14^ (or its counterpart, adaptation) where the initial response of the system is higher than the steady-state level, or, from another perspective, the system has the ability to produce a stronger response than that predicted by the steady-state, but only for a restricted amount of time. This effect is analogous to what is called adaptation in sensory and biochemical systems, and resembles an overshot in the temporal response.

Signal termination is a relatively recent finding in CMCs, which has been observed in the enzyme-in-excess regime, but not in the substrate-in-excess regime, which has been the most studied so far. Periodic stimulation of CMCs serves the purpose of understanding how these systems react to time-varying inputs. On the one hand, periodic stimulation is arguably the simplest type of non-decaying (with time) input. On the other hand, the response to sinusoidal inputs can be used to reconstruct the response signal to a rather general class of time-varying inputs. Square wave inputs constitute a link between both the step-constant and sinusoidal inputs where the input is periodic and changes abruptly between two constant values, thus periodically reproducing the transient effects described above in a frequency-dependent manner. The response of CMCs to periodic stimulation has been primarily studied in the substrate-in-excess regime where the product responses have been shown to be low-pass filters. However, outputs that terminate in response to step-constant inputs have been linked to band-pass filter responses to sinusoidal inputs^24–27^, thus raising the possibility that CMCs in the enzyme-in-excess regime exhibit this type of responses to periodic inputs in general, and both signal termination and preferred frequency responses are communicated across CMCs in the cascades of which they are part. In this paper, we set out to address these issues via a combined modeling and computational approach.

It is common practice to consider that the number of enzymes, either kinase, or the ratio between kinase and phosphatase, drive the activity in a CMC^3,10,28,41^. This is typically done in the substrate-in-excess regime. However, in this article, we considered the opposite, enzyme-in-excess regime. In this case, stimulating the enzyme levels produces almost no effect as shown in Supplementary Figure 1). We then stimulated the system by changing the amount of substrate, particularly, the total level of the substrate. It is also important to notice that in several cases substrates are enzymes as well. This is the case of kinase cascades in which the activated substrate in one level works as the enzyme for the downstream level. The MAPK cascade is a well-known example in this regard^10^. In these cascades, stimulating the enzymes or the substrates could play interchangeable roles.

The total amount of proteins in a CMC usually changes in a slower time scale than that of signaling, but that is not always the case and several studies consider total variations in the same time scale as that of signaling^29,39^. The effect of regulating the total level of a signaling protein and the impact this could have in its dynamics was comprehensively studied both theoretically and computationally^22,42^. Even when experimental results are available in the analogous ligand–receptor reaction scenario, the common practice is to consider that the ligand is the stimulus. However, in certain articles, it was convenient to stimulate the receptor level^43^.

In the same way, the total amount of substrate was treated as a signal in this paper, other factors can be treated as a signal too. In Supplementary Figure 2 the signal is *k*_*1*_, the kinase catalytic rate. A step-like variation in *k*_*1*_ leads to a signal termination response, while a periodic forcing in *k*_*1*_ leads to a frequency-preference response. Similar studies are found in the literature considering different factors as a signal, as the substrate unbinding rate^44^, the kinase inactivation rate^45^, and the degradation rate^46^, to mention some examples.

The stimulation selected in this study, the total substrate concentration, places this article among those evaluating absolute concentration robustness (ACR)^47,48^. ACR implies that the steady-state output of a system is completely independent of a protein concentration. For CMCs with high enzyme concentration, i.e., CMCs in the enzyme-in-excess regime, it was reported that concentration robustness only occurs under certain conditions with respect to total enzyme concentrations^6^. Even though we have not evaluated theoretically ACR in the enzyme-in-excess regime with respect to total substrate concentration, the results we obtained indicate that it does not occur. The reason for this is that systems with ACR have robust perfect adaptation for the target species. A system possesses perfect adaptation if the output of the system returns to the pre-stimulus level after the value of the input parameter is changed to a different constant level^49–53^. If the system achieves perfect adaptation independently of the system parameters, it is said to have robust perfect adaptation. As shown in this article, after the transient response the system does not generally return to pre-stimulation levels. In summary, CMCs in the enzyme-in-excess regime exhibit neither perfect adaptation nor ACR with respect to total substrate concentration in a purely kinetic framework. However, it could be possible that when considering spatial organization, even though the total enzyme may be in excess, the dynamics might be different locally^54^.

Motivated by the predictions in^14^, we characterized the signal termination responses in terms of a number of attributes (peak *P*_max_ and steady-state *P*_ss_) and excluded from the analysis those cases for which signal termination is mild (small *P*_max_, low *P*_max_/*P*_ss_). The key elements for the signal to terminate are the relative amounts of enzymes and substrate, the relative velocities of the two modifying enzymes, and their relative affinities. The underlying mechanism is relatively simple, when the substrate is added in a step-like profile, a fast kinase modifies it producing the product, while a slower phosphatase undergoes the opposite process terminating the signal. Our results (Figs. 1–3) quantitatively confirmed the conditions stated in (Bluthgen et al., 2006), namely that if the enzymes are in large excess over the substrate, if the kinase is fast and has low affinity, and if the phosphatase is slow and has high affinity, then signal termination occurs. We referred to them as the *Bluthgen conditions*.

To more deeply understand and further characterize signal termination we combined analytical calculations, local analysis, and a parameter space exploration. Some of the studies were performed over specific, representative parameter sets (Figs. 1, 5, and 7), whereas others are based on this parameter space exploration (Figs. 3, 6, and 8). The representative parameter sets capture scenarios that have more general validity. For this first group, we included the results of alternative parameter sets in the Supplementary Information, showing that the conclusions extracted from those studies are independent of the choice of parameter set (Supplementary Section 5).

The goals of the parameter space exploration were: (i) to find out the connection between parameter sets satisfying Bluthgen conditions and outputs exhibiting signal termination (according to the criteria defined in Section 1); (ii) to investigate whether signal termination persists if any of the four Bluthgen conditions (*E*_*1T*_/*S*_*T*_ > 1, *E*_*2T*_/*S*_*T*_ > 2, *V*_*1*_/*V*_*2*_ > 1, *Aff*_*2*_/*Aff*_*1*_ > 1) are relaxed, which one can be relaxed, to what extent, and which exert tighter control over signal termination; and iii) to understand which parameter set characteristics lead to strong signal termination. Strong signal termination is a more restrictive condition in which the signal terminates to almost pre-stimulation levels, close to what is known as perfect adaptation.

Although signal termination arises in an enzyme-in-excess regime, our results show that this is not a necessary condition, but signal termination can occur in a regime where the enzymes and the substrate are in similar (comparable) amounts. Particularly, the phosphatase level has a tighter control on signal termination, being this enzyme is the one that converts back the product into the unmodified substrate thus terminating the signal. The velocities and affinities requirements (*V*_*1*_ > *V*_*2*_ and *Aff*_*2*_ > *Aff*_*1*_) can be relaxed while still observing signal termination, but one at a time. In fact, relaxing both together leads to a monotonic temporal profile. If condition *E*_*2T*_/*S*_*T*_ > 1 is not satisfied, conditions *Aff*_*2*_/*Aff*_*1*_ and *V*_*1*_/*V*_*2*_ are both required, instead, if it is condition *E*_*1T*_/*S*_*T*_ > 1 that is not satisfied, only one extra condition (*Aff*_*2*_/*Aff*_*1*_ or *V*_*1*_/*V*_*2*_) can lead to signal termination (this conclusion is extracted from Fig. 3c).

Before we could determine whether the parameter sets leading to signal termination under constant stimulation ensured a preferred frequency response under periodic stimulation, we needed to overcome an important issue, which is common to any system in which the quantity that needs to be controlled satisfies a conservation law and is under sequestration conditions: *S*_*T*_ cannot follow the desired periodic profile, an adapted but still, periodic one was used instead (Fig. 4). This must be done so as not to include negative values for amounts of the participating species. Specifically, the chemical species at play cannot follow any desired periodic profile, the time scale involved in the release from sequestration together with the forcing time scale determine an adapted stimulation profile. This led to a modification of the stimulation protocol that sacrificed uniformity across frequencies in the input for the sake of understanding the preferred frequency properties of CMCs under realistic conditions and appropriate normalization that made the comparisons across frequencies possible.

The adaptation of the input signal is a necessity dictated by the system and not our choice. Our choices lie simply in (1) the type of adaptation among more than one possible option, and (2) the choice of a system that presents this problem. One solution to the latter would have been to avoid the study of this system and choose a “more elegant one”. However, we believe we are shedding light on a problem that presents itself in realistic situations. To our knowledge, frequency-preference studies of complex enzymatic reactions are not abundant in the literature, most likely because of the type of issues we are discussing here. In the cases where results were successfully obtained without the need for adaptation in the input signal, it is not clear whether all the variables remain positive or only the variables presented in the graphs and whether the model reductions used conserve all the frequency-dependent information present in the full model from where they originate. To our knowledge, these issues have not been discussed in the literature on CMCs and related biochemical systems, and call for further research and examination.

The main idea we test in this paper is whether there is a relationship between signal termination and the amplitude profile properties of CMCs to periodic inputs. This knowledge allows inferring the presence of time scales that support these phenomena. While engineering studies of linear systems focus on prediction, other scientific studies (e.g., cell signaling and neuroscience) focus on inferring the presence of time scales that are common to different, apparently disconnected phenomena. In this paper, we follow this tradition. We acknowledged and discussed in detail the difficulties in applying a uniform protocol across frequencies. However, by normalizing by the input amplitude we overcome this issue, if not totally, partially, but this does not affect our ability to learn from the system what we intended to learn. Furthermore, Supplementary Figure 4 shows that the gain is not affected by the amplitude of the input, a result that was expected by the definition of gain. In addition, the responses measured in terms of the standard impedance (quotient of the Fourier transforms of the output and the input, in absolute value) have predictive value, as indicated in Supplementary Figure 5.

Regarding which biological situation could lead to such a periodic control of the total amount of substrate, it is well-known that signaling proteins are promiscuous, i.e., the same protein is shared by different pathways leading in several cases to a specificity problem^55,56^. The use of the free amount of protein by others pathways, particularly oscillating pathways could lead to the situation described in this article. Although we agree, these pathways will most likely not lead to nice sinusoidal or square wave inputs, these are, in our opinion, the most sensible ones for an organized study of the phenomena.

We evaluated the existence of preferred (optimal) frequency responses to periodic stimulation (band-pass filters) in terms of two quantities that provide complementary information: amplitude and gain. We used the two types of input protocols mentioned above, which also provide complementary information: sinusoidal and square waves. We found that the CMC exhibited preferred frequency responses in the gain for parameter values for which the underlying autonomous system exhibits signal termination, whereas the output amplitude did not necessarily capture the optimal response, and this depended on the type of input protocol. Analyzing correlation graphs between the attributes for signal termination and corresponding preferred frequency responses, we found that the effective slower time scales of the autonomous CMCs played a significant role in determining their preferred responses to sinusoidal inputs. These results indicated that input is more effective in producing a significant output if it allows the system to terminate the signal before the new stimulation cycle begins. These results are consistent with previous findings in neuronal systems^24^.

From the biochemical point of view, the preferred frequency response in the gain profile can be generated by two different mechanisms as the relevant biochemical parameters change (Fig. 7): (i) *G* (gain) increases faster for intermediate frequencies than for lower frequencies, and (ii) *G* decreases faster for lower frequencies than for intermediate frequencies. These results together with the results discussed above strengthen the relationship between the emergence of preferred frequency response to periodic inputs and signal termination beyond the parameter exploration exercise.

Testing the hypothesis that signal termination and preferred frequency response to oscillatory inputs in isolated CMCs play a role for the responses of the networks in which these cycles are embedded, to both step-constant and periodic stimulation, requires the identification of representative case studies to avoid the rapid explosion of parameter combinations. In this paper, we focused on the minimal network model consisting of a cascade of two CMCs and identified five cases studies that allowed us to evaluate whether the signal is affected by a downstream cycle and if it can be propagated downstream in the cascade (Fig. 8, Study 1), if this behavior can emerge from the coupling of cycles as a network effect (Fig. 8, Studies 2–5), and if new behavior can emerge from the coupling of CMCs. Within the explored ranges we found no new dynamic behavior, contrary to our expectation of uncovering damped oscillations^7^. We concluded that signal termination can be propagated to a downstream cycle and that this second cycle is not able to remove the signal termination in the upper one.

Interestingly, we found that a monotonic behavior in the first cycle can be transformed into signal termination by the addition of a second cycle with particular parameter values (Fig. 8, Study 2, output i). The opposite study, i.e., sampling on the upper cycle, is not able to remove signal termination in the downstream one (Fig. 8, Study 3), reinforcing that the control of the behavior is exerted by the phosphatase of the cycle with signal termination, cycle 2 in the considered study. These studies on cascades were complemented with the search of preferred frequency responses and the analysis of amplification and attenuation in the cascade (Fig. 9).

The nature of the perturbation of the first cycle by the second that alters the first cycle’s behavior is called retroactivity, i.e, the modulation of the first cycle’s behavior performed by its load, the second cycle^7,17,18,57^. This change of behavior can have different effects depending on the parameter conditions.

The discussion on simplifying assumptions for modeling multi-step pathways in systems biology is very active in the literature^58^. Based on it, one would wonder if it is possible to attempt some theoretical analysis of the response of the system. As we see it, this type of analysis could go in three directions: (i) reducing the dimensionality of the system to two dimensions, (ii) linearizing the system and preserving the three dimensions, or (iii) developing tools to analyze the original 3D system. The reduction of dimensionality necessarily needs to be based on the identification of a separation of time scales and keeping only the portion with slow dynamics. But, we in fact show in the paper that the relevant dynamics are lost if we proceed in this way (Section 4, signal termination and frequency-preference response are not captured by usual CMC’s approximations). Linearizing the system around the equilibrium would produce the expected results about signal termination and resonance, but its validity will be restricted to a very small region in the phase-space around this equilibrium, and therefore it would not be a reasonable analytical approach to explain our results. The development of analytical tools to analyze the original 3D system is the most intellectually tempting option and there are various possible approaches. One of them is using piecewise linear systems and analyzing the evolution of the trajectory as it crosses regimes with different combinations of linear pieces. Another one is to track the responses of (“moving”) 3D phase-space diagrams in response to the inputs with the aid of the graphs of null surfaces. Both of these approaches are complex and are beyond the scope of the present paper.

An important feature of our study is that our (mechanistic) modeling approach did not use the available and ubiquitous approximations, which often render one-dimensional models that are expected to be unable to produce signal termination and preferred frequency responses to oscillatory inputs. To confirm this and to further understand the effects of the removal of the fast time scales from the models by the application of the dimensionality reduction processes, we repeated some of the protocols using the reduced approximated models. As expected, the tQSSA produced no signal termination and low-pass filtering. The other approximations considered did produce signal termination and preferred frequency response to periodic inputs, but not good approximations to similar results using the mechanistic model (Fig. 10). Analogous consequences are found in other areas, in which the search of simple and compact models leads to dimensionality reduction that precludes non-monotonic temporal profiles such as the ones described in this article^59,60^.

Different network topologies can lead to an overshoot transient response. The incoherent feedforward loop is one of them^19^. However, the ability of a CMC to produce similar transient responses was only studied by Bluthgen and colleagues. Furthermore, the groundbreaking article by Ma and colleagues defining network topologies that can achieve biochemical adaptation^19^, considers three-node topologies where each node is a CMC. This indicates that the literature considers much more complicated systems than an isolated CMC in order to explain overshoot/signal termination/adaptation responses. The unstated implication is that these topologies are the minimal ones that produce the phenomenon. We reasoned that if the simple two-dimensional linear systems are able to show signal termination, then CMCs should also be able to display this phenomenon, at least in a regime where the dynamics unfold in more than one effective dimension and the necessary time scales are preserved. In fact, our studies indicate that the reason behind the current understanding in the literature is twofold: (1) CMCs were mostly studied in the substrate-in-excess regime, where signal termination does not occur, (2) signal termination is not captured by the usual approximations, as they rely on the removal of the fast time scales. These observations partly explain why signal termination and frequency-preference response were overlooked in this ubiquitous signaling motif.

Regarding the modeling approach in this article, there are instances where modification cycle/cascades may not be described in ODE terms and these include various contexts where the spatial dimension is important^61^. This issue requires more research.

Summarizing, substrate sequestration by its modifying enzymes, and in particular by the phosphatase, might be a means to achieve signal termination and desensitization downstream of receptors without involving an explicit negative feedback loop. This behavior can be at play under physiological conditions, as the abundances of substrates and enzymes are similar in vivo. Therefore, it is relevant to study how CMCs, cascades of CMCs, and signaling pathways combining them, process different temporal inputs under sequestration conditions. Our predictions about the band-pass filter behavior of these elements (when periodically stimulated) shed interesting insights into fundamental biological processes and the computations that might be carried in biochemical networks. More research is needed to test these predictions experimentally and to examine the functional consequences of band-pass filter behavior in CMCs for larger networks in which CMCs are embedded. Although preferred frequency responses of CMCs could be an epiphenomenon, research in other areas shows they may be functional for the generation of network oscillations^62,63^.

## Methods

All the algorithms used in this paper were written in Python 3.8, Spyder 4. The libraries used were numpy, numba, pyDOE, pylab.

### Mechanistic description for a CMC

Following the scheme, reactions, variables, and parameters indicated in Fig. 1a and using the Law of Mass Action, the ode system describing a CMC is the following:

$$\frac{{\rm{d}}{C_1}}{\rm{dt}} = a_1E_1S - \left( {d_1 + k_1} \right)C_1$$

$$\frac{{\rm{d}}{C_2}}{\rm{dt}} = a_2E_2P - \left( {d_2 + k_2} \right)C_2$$

$$\frac{{\rm{d}}{P}}{\rm{dt}}= k_1C_1 - a_2PE_2 + d_2C_2,$$

combined with the following conservations laws:

$$E_{1T} = E_1 + C_1;\;E_{2T} = E_2 + C_2;\;S_T = P + S + C_1 + C_2$$

### Mechanistic description for a cascade with two CMCs

Following the scheme, reactions, variables, and parameters indicated in Fig. 8a and using the Law of Mass Action, the ode system describing a two-level cascade is the following:

$$\frac{{\rm{d}}{C_1}}{\rm{dt}} = a_1E_1S_1 - \left( {d_1 + k_1} \right)C_1$$

$$\frac{{\rm{d}}{C_1^\prime}}{\rm{dt}}= a_1^\prime E_1^\prime P_1 - \left( {d_1^\prime + k_1^\prime } \right)C_1^\prime$$

$$\frac{{\rm{d}}{C_2}}{\rm{dt}}= a_2P_1S_2 - \left( {k_2 + d_2} \right)C_2$$

$$\frac{{\rm{d}}{C_2^\prime}}{\rm{dt}}= a_2^\prime E_2^\prime P_2 - \left( {k_2^\prime + d_2^\prime } \right)C_2^\prime$$

$$\frac{{\rm{d}}{P_1}}{\rm{dt}}= k_1C_1 - a_1^\prime P_1E_1^\prime + d_1^\prime C_1^\prime - a_2P_1S_2 + \left( {d_2 + k_2} \right)C_2$$

$$\frac{{\rm{d}}{P_2}}{\rm{dt}}= k_2C_2 - a_2^\prime E_2^\prime P_2 + d_2^\prime C_2^\prime$$

combined with the following conservations laws:

$$E_{1T} = E_1 + C_1;\;E_{1T}^\prime = E_1^\prime + C_1^\prime ;\;E_{2T}^\prime = E_2^\prime + C_2^\prime$$

$$S_{1T} = S_1 + P_1 + C_1 + C_1^\prime + C_2;\;S_{2T} = S_2 + P_2 + C_2 + C_2^\prime$$

### Parameters definitions and units

*a*, association rate

*d*, dissociation rate

*k*, catalytic rate

*E*_*T*_, total amount of kinase

*E*_*T*_’, total amount of phosphatase

*S*_*T*_, total substrate

Rates *a*, *d, k* could appear without or with an apostrophe, indicating that they are associated with the reaction catalyzed by the kinase or the phosphatase, respectively. Those rates could appear with a subindex 1 or 2, indicating the first and second cycle in the case of a cascade. For the numerical simulations, the initial concentrations are such that all the substrate is in the unmodified form and all the enzymes are in the free form (i.e., are not forming complexes). The values selected for the parameters are indicated in each figure and summarized in Supplementary Table 1.

Some of the parameters have dimensions of concentration (*E*_*T*_, *E*_*T*_*’*, *S*_*T*_), some have dimensions of 1/time (*d*, *k*), and others have dimensions of 1/(time **·** concentration) (*a*). The unit of time is selected as minute (so that the duration of the responses in signal termination is consistent with significant biological durations), and the unit of concentration is arbitrary. This means that when a value for a parameter is listed, the corresponding unit has to be added, for example, if the value of parameter a is 10, it has to be read as 10 × 1/(min × concentration). Once the reference concentration is selected, all the parameters follow that reference unit. The interpretation of the results depends on the choice of the reference unit concentration (the ‘0’ in log scale). For example, if the reference dimensional concentration is chosen as 0.1 μM, this leads to interpreting the scanned intervals (from 10^1^ to 10^2^) as being in the range (1 μM, 10 μM). However, this is just an example and the choice of the reference unit concentration remains a degree of freedom in our numerical methodology.

### Parameter space exploration for signal termination

We took as an initial guide for parameter values and ranges, the intervals obtained from the literature and used in previous articles^40,64^ and the table published in the Supplementary information of Bluthgen’s article^14^. The first intervals indicated a range of 0.3–224 for the enzymes and 3–1000 for the substrate, evidencing that they were based on a substrate-in-excess regime. We modified them for this article so that both enzymes and the substrate take values in the same range, 10–100. *k*_1_, *k*_2_ are in the range 1–50, which is similar to that in the mentioned intervals (6.3–600). Parameters *a*_1_, *d*_1_, *a*_2_, *d*_2_ were selected in the range 0.1–10 according to Bluthgen’s table. Every parameter set in the paper was taken from these ranges.

We logarithmically sampled parameters *a*_1_, *d*_1_, *a*_2_, *d*_2_ between 10^−1^ and 10^1^, *k*_*1*_, *k*_*2*_ between 1 and 50, and *S*_*T*_, *E*_*1T*_, *E*_*2T*_ between 10^1^ and 10^2^ using Latin Hypercube Sampling^65^ for 20,000 different sets. To do that, we used pyDOE Python library and sampled linearly in the exponents. We scanned the 20,000 sets, dividing the process into four independent routines of 5,000 sets of different samples.

### Parameter space exploration for frequency preference

The frequency response was studied in 5,000 parameters sets. Each set was stimulated with 10 different frequencies *f*, equally spaced logarithmically in the range 10^–2^/60–10–1/60, with step 10^0.1^/60. In each case, gain and amplitude were measured. If the profile obtained with these ten data points indicates an increasing function, it is concluded that it will exhibit a frequency preference, so *f* is increased until finding a decreasing trend. In this process, the preferred frequency is obtained. If instead, the profile (gain or amplitude versus frequency) shows a decreasing trend for all the explored frequencies, it is assumed that this is a low-pass filter. *G*_*0*_ always corresponds to *G*_*0*_=*G*(*f* = 10^−2^/60). Once a frequency-preference case is identified, a finer grid is used to better capture *f*_max_ (*f* is varied between 0.5*f*_max_ and 2*f*_max_ with a step of 0.05*f*_max_). With this procedure, *G*_max_ and *f*_max_ are estimated again. This process is repeated twice.

### Analysis of the parameter space exploration for cascades

The outputs of the five studies performed with cascades are analyzed in the following way. The criteria to identify signal termination is adapted from Section 1, being more flexible for cascades: levels 1 or 2 are said to have signal termination if the maximum of the temporal profile is higher than 0.1 and *Q* is higher than 1.2 (given that *Q* = *P*_max_/*P*_ss_, this condition means that there must be a decrease of at least 0.83% from the maximum to the steady-state to classify the output as having signal termination).

If *P*_*1*_ and *P*_*2*_ do not satisfy the criteria for signal termination, this set is labeled as monotonic for both cycles. If *P*_*1*_ or *P*_*2*_ (only one of them) satisfies the criteria for signal termination, then this set is labeled as signal termination in the first or second cycle, respectively. If, instead, both *P*_*1*_ and *P*_*2*_ satisfy the criteria, this set is labeled as signal termination in both cycles. Outputs, where *P*_*1*_ and *P*_*2*_ are not monotonic but do not reach the criteria for signal termination, are not considered for the analysis.

Frequency preference is evaluated in the cycle that exhibits signal termination, if only one of them does it, and in the second cycle if both of them have signal termination.

### Procedure to obtain an adapted periodic input

The adapted sinusoidal input is defined as follows:

*S*_*T*_ = *S*_*T,*max_ (1 + 0.5sin (2π*ft*))/1.5and the adapted train of square pulses is defined as follows:

*S*_*T*_ = *S*_*T,*max_ if mod(*t*,*T*) < *d*, *S*_*T*_ = 0 if not, where *f* is the frequency, mod means module, *T* is the period, and *d* is the length of the square. In this way, and given *S*_*T,*desired_ the sinusoidal input or the train of square pulses, we adapt *S*_*T*_ so that it is always positive. We do so by changing free substrate value S in this way: if *S* = *S*_*T*desired_-*P*-*C*_*1*_-*C*_*2*_ < 0, then *S* = 0.

### Reporting summary

Further information on research design is available in the Nature Research Reporting Summary linked to this article.

## Supplementary information

Supplementary Information

Reporting Summary

## Data Availability

The data in the figures were obtained with codes prepared in Python 3.8, Spyder 4. Data are available upon email request.

## Code availability

The data in the figures were obtained with codes prepared in Python 3.8, Spyder 4. Source codes are available upon email request.

## References

[1] Goldbeter A, Koshland DE (1981). An amplified sensitivity arising from covalent modification in biological systems. Proc. Natl Acad. Sci. USA.

[2] Alberts B., et al. *Molecular Biology of the Cell*. (Garland Science, 2001).

[3] Gomez-Uribe C, Verghese GC, Mirny LA (2007). Operating regimes of signaling cycles: statics, dynamics, and noise filtering. PLoS Comput. Biol..

[4] Kholodenko B (2006). Cell-signalling dynamics in time and space. Nat. Rev. Mol. Cell Biol..

[5] Markevich NI, Hoek JB, Kholodenko BN (2004). Signaling switches and bistability arising from multisite phosphorylation in protein kinase cascades. J. Cell Biol..

[6] Straube R (2017). Operating regimes of covalent modification cycles at high enzyme concentrations. J. Theor. Biol..

[7] Ventura AC, Sepulchre J-A, Merajver SD (2008). A hidden feedback in signaling cascades is revealed. PLoS Comput. Biol..

[8] Albe KR, Butler MH, Wright BE (1990). Cellular concentrations of enzymes and their substrates. J. Theor. Biol..

[9] Aragón JJ, Sols A (1991). Regulation of enzyme activity in the cell: effect of enzyme concentration. FASEB J..

[10] Huang CYF, Ferrell JE (1996). Ultrasensitivity in the mitogen-activated protein kinase cascade. Proc. Natl Acad. Sci. USA.

[11] Martins, B. M. C. & Swain, P. S. Ultrasensitivity in phosphorylation-dephosphorylation cycles with little substrate. *PLoS Comput. Biol*. **9**, e1003175 (2013). 10.1371/journal.pcbi.1003175 PMC3738489 23950701

[12] Schnell S, Maini PK (2000). Enzyme kinetics at high enzyme concentration. Bull. Math. Biol..

[13] Choi B, Rempala GA, Kim JK (2017). Beyond the Michaelis-Menten equation: accurate and efficient estimation of enzyme kinetic parameters. Sci. Rep..

[14] Bluthgen N (2006). Effects of sequestration on signal transduction cascades. FEBS J..

[15] Mangan S, Zaslaver A, Alon U (2003). The coherent feedforward loop serves as a sign-sensitive delay element in transcription networks. J. Mol. Biol..

[16] Legewie S, Schoeberl B, Blüthgen N, Herzel H (2007). Competing docking interactions can bring about bistability in the MAPK cascade. Biophys. J..

[17] Jiang P (2011). Load-induced modulation of signal transduction networks. Sci. Signal..

[18] Ventura AC (2010). Signaling properties of a covalent modification cycle are altered by a downstream target. Proc. Natl Acad. Sci. USA.

[19] Ma W, Trusina A, El-Samad H, Lim WA, Tang C (2009). Defining network topologies that can achieve biochemical adaptation. Cell.

[20] Ferrell JE (2016). Perfect and near-perfect adaptation in cell signaling. Cell Syst..

[21] Ventura AC (2014). Utilization of extracellular information before ligand-receptor binding reaches equilibrium expands and shifts the input dynamic range. Proc. Natl Acad. Sci. USA.

[22] Soyer OS, Kuwahara H, Csikász-Nagy A (2009). Regulating the total level of a signaling protein can vary its dynamics in a range from switch like ultrasensitivity to adaptive responses. FEBS J..

[23] Lemmon MA, Freed DM, Schlessinger J, Kiyatkin A (2016). The dark side of cell signaling: positive roles for negative regulators. Cell.

[24] Rotstein HG (2014). Frequency preference response to oscillatory inputs in two-dimensional neural models: a geometric approach to subthreshold amplitude and phase resonance. J. Math. Neurosci..

[25] Rotstein HG, Nadim F (2014). Frequency preference in two-dimensional neural models: a linear analysis of the interaction between resonant and amplifying currents. J. Comput. Neurosci..

[26] Richardson MJE, Brunel N, Hakim V (2003). From subthreshold to firing-rate resonance. J. Neurophysiol..

[27] Wilson MZ, Ravindran PT, Lim WA, Toettcher JE (2017). Tracing information flow from Erk to target gene induction reveals mechanisms of dynamic and combinatorial control. Mol. Cell.

[28] Cournac A, Sepulchre J (2009). Simple molecular networks that respond optimally to time-periodic stimulation. BMC Syst. Biol..

[29] Di Talia S, Wieschaus EF (2014). Simple biochemical pathways far from steady state can provide switchlike and integrated responses. Biophys. J..

[30] Fletcher, P. A., Clément, F., Vidal, A., Tabak, J. & Bertram, R. Interpreting frequency responses to dose-conserved pulsatile input signals in simple cell signaling motifs. *PLoS ONE* **9**, e95613 (2014). 10.1371/journal.pone.0095613 PMC3991699 24748217

[31] Keener, J. P. & Sneyd, J. *Mathematical Physiology*. (2008). 10.1007/978-0-387-79388-7

[32] Veuthey AL, Stucki J (1987). The adenylate kinase reaction acts as a frequency filter towards fluctuations of ATP utilization in the cell. Biophys. Chem..

[33] Schoch A, Pahle J (2019). Requirements for band-pass activation of Ca 2+ -sensitive proteins such as NFAT. Biophys. Chem..

[34] Sumit M, Neubig RR, Takayama S, Linderman JJ (2015). Band-pass processing in a GPCR signaling pathway selects for NFAT transcription factor activation. Integr. Biol. (Camb.)..

[35] Hanson SM, Schnell S (2008). Reactant stationary approximation in enzyme kinetics. J. Phys. Chem. A.

[36] Eilertsen J, Stroberg W, Schnell S (2019). Characteristic, completion or matching timescales? An analysis of temporary boundaries in enzyme kinetics. J. Theor. Biol..

[37] Rotstein HG (2015). Subthreshold amplitude and phase resonance in models of quadratic type: nonlinear effects generated by the interplay of resonant and amplifying currents. J. Comput. Neurosci..

[38] Tzafriri AR (2003). Michaelis-Menten kinetics at high enzyme concentrations. Bull. Math. Biol..

[39] Di Talia S, Wieschaus EF (2012). Short-term integration of Cdc25 dynamics controls mitotic entry during drosophila gastrulation. Dev. Cell.

[40] Di-Bella JP, Colman-Lerner A, Ventura AC (2018). Properties of cell signaling pathways and gene expression systems operating far from steady-state. Sci. Rep..

[41] Levine J, Hao YK, Mirny L (2007). Intrinsic fluctuations, robustness, and tunability in signaling cycles. Biophys. J..

[42] Sepulchre, J. A., Merajver, S. D. & Ventura, A. C. Retroactive signaling in short signaling pathways. *PLoS One* **7**, e40806 (2012). 10.1371/journal.pone.0040806 PMC3406091 22848403

[43] O’Donoghue, G. P. et al. T cells selectively filter oscillatory signals on the minutes timescale. *Proc. Natl. Acad. Sci. USA* **118**, (2021). 10.1073/pnas.2019285118 PMC7936380 33627405

[44] Reuveni S, Urbakh M, Klafter J (2014). Role of substrate unbinding in Michaelis-Menten enzymatic reactions. Proc. Natl Acad. Sci. USA.

[45] Behar M, Hoffmann A (2013). Tunable signal processing through a kinase control cycle: the IKK signaling node. Biophys. J..

[46] Di Talia S (2013). Posttranslational control of Cdc25 degradation terminates drosophila’s early cell-cycle program. Curr. Biol..

[47] Shinar G, Feinberg M (2010). Structural sources of robustness in biochemical reaction networks. Science.

[48] Batchelor E, Goulian M (2003). Robustness and the cycle of phosphorylation and dephosphorylation in a two-component regulatory system. Proc. Natl Acad. Sci. USA.

[49] Ang J, Bagh S, Ingalls BP, McMillen DR (2010). Considerations for using integral feedback control to construct a perfectly adapting synthetic gene network. J. Theor. Biol..

[50] Ang J, McMillen DR (2013). Physical constraints on biological integral control design for homeostasis and sensory adaptation. Biophys. J..

[51] Drengstig T, Ueda HR, Ruoff P (2008). Predicting perfect adaptation motifs in reaction kinetic networks. J. Phys. Chem. B.

[52] Krishnan J, Floros I (2019). Adaptive information processing of network modules to dynamic and spatial stimuli. BMC Syst. Biol..

[53] Thorsen, K. et al. Robust concentration and frequency control in oscillatory homeostats. *PLoS ONE* **9**, e107766 (2014). 10.1371/journal.pone.0107766 PMC4169565 25238410

[54] Krishnan, J., Lu, L. & Nazki, A. A. The interplay of spatial organization and biochemistry in building blocks of cellular signalling pathways. *J. R. Soc. Interface* **17**, 20200251 (2020). 10.1098/rsif.2020.0251 PMC7276544 32453980

[55] Komarova NL, Zou X, Nie Q, Bardwell L (2005). A theoretical framework for specificity in cell signaling. Mol. Syst. Biol..

[56] Rowland MA, Harrison B, Deeds EJ (2015). Phosphatase specificity and pathway insulation in signaling networks. Biophys. J..

[57] Del Vecchio, D., Ninfa, A. J. & Sontag, E. D. Modular cell biology: retroactivity and insulation. *Mol. Syst. Biol*. **4**, 161 (2008). 10.1038/msb4100204 PMC2267736 18277378

[58] Korsbo N, Jönsson H (2020). It’s about time: Analysing simplifying assumptions for modelling multi-step pathways in systems biology. PLoS Comput. Biol..

[59] Fernandez-Lopez, R., del Campo, I., Revilla, C., Cuevas, A. & de la Cruz, F. Negative feedback and transcriptional overshooting in a regulatory network for horizontal gene transfer. *PLoS Genet*. **10**, e1004171 (2014). 10.1371/journal.pgen.1004171 PMC3937220 24586200

[60] Val-Calvo J (2018). Novel regulatory mechanism of establishment genes of conjugative plasmids. Nucleic Acids Res..

[61] Alam-Nazki A, Krishnan J (2015). Spatial Control Of Biochemical Modification Cascades And Pathways. Biophys. J..

[62] Bel, A. & Rotstein, H. G. Membrane potential resonance in non-oscillatory neurons interacts with synaptic connectivity to produce network oscillations. *J. Comput. Neurosci*. **46**, 169–195 (2019). 10.1007/s10827-019-00710-y 10.1007/s10827-019-00710-y 30895410

[63] Chen Y, Li X, Rotstein HG, Nadim F (2016). Membrane potential resonance frequency directly influences network frequency through electrical coupling. J. Neurophysiol..

[64] Ossareh HR, Ventura AC, Merajver SD, Del Vecchio D (2011). Long signaling cascades tend to attenuate retroactivity. Biophys. J..

[65] McKay MD, Beckman RJ, Conover WJ (2000). A comparison of three methods for selecting values of input variables in the analysis of output from a computer code. Technometrics.

