# Supplementary material for: Frequency-preference response in covalent modification cycles under substrate sequestration conditions
Source: NPJ Syst Biol Appl. 2021 Aug 17;7:32. doi: 10.1038/s41540-021-00192-8 (PMC8371027; doi:10.1038/s41540-021-00192-8)
Supplement: Supplementary file 1 — Supplementary Information [file 41540_2021_192_MOESM1_ESM.pdf]

Supplementary Information

Frequency preference response in covalent modification cycles under sequestration conditions

Juliana Reves Szemere, Horacio G Rotstein, Alejandra C Ventura

1. Total substrate as a signal.

We include here two figures related to the decision of stimulating through S_T, the total substrate concentration.

Supplementary Fig. 1 shows the results of stimulating through E_{1T}, the total kinase concentration, which is in excess. As we see in Supplementary Fig. 1A, there is no need of adapting E_{1T} as is the case with S_T (Fig. 4, main text), Supplementary Fig. 1B indicates that almost all the kinase is in its free form. Supplementary Fig. 1C shows the almost negligible effect the stimulating the variable that is in excess has on the product

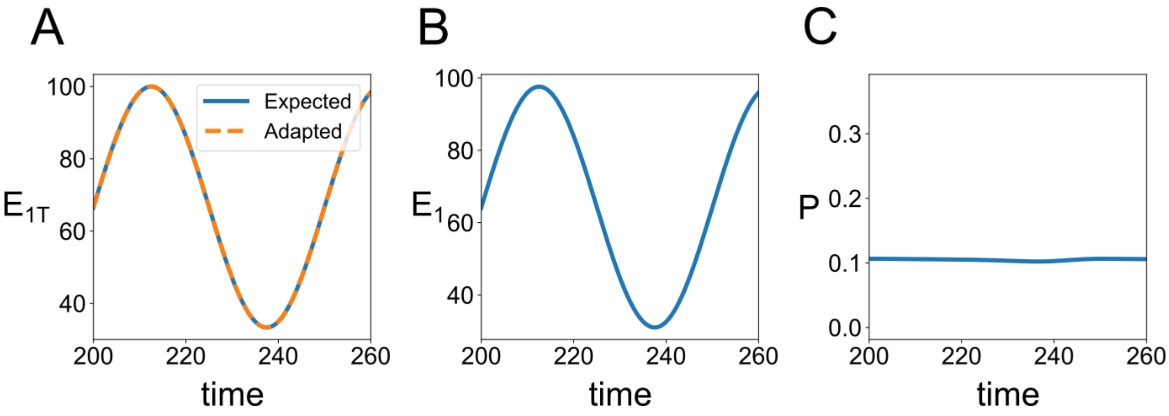

Supplementary Figure 1. Stimulation in E_{1T} (total kinase). A. Expected and adapted variations in E_{1T}. **B.** E_1 free form temporal profile. **C.** Product temporal profile. Parameter values: a_1 = 3.5; d_1 = 1; k_1 = 30; a_2 = 0.3; d_2 = 0.25; k_2 = 2.5; E_{1T} = 100; E_{2T} = 100; S_T = 35. The frequency of stimulation is f=0.02Hz, corresponding to T=50s.

Supplementary Fig. 2 shows the results of stimulating the rate k_1, the kinase catalytic rate, instead of stimulating S_T as done in the main text. The numerical simulation starts with the initial condition S_T=0 and using the same parameters values as in Fig. 1, except for k_1. A step in S_T is applied. After the system has reached steady-state, at t=100 s, k_1 is varied in a step-like manner. The step in k_1 produces a signal termination profile in P (Supplementary Fig. 2A) and a preferred frequency response both in amplitude and in gain (Supplementary Fig. 2B and C). The results are not conclusive, but it is a proof of principle of the generality of our results for other parameter perturbations.

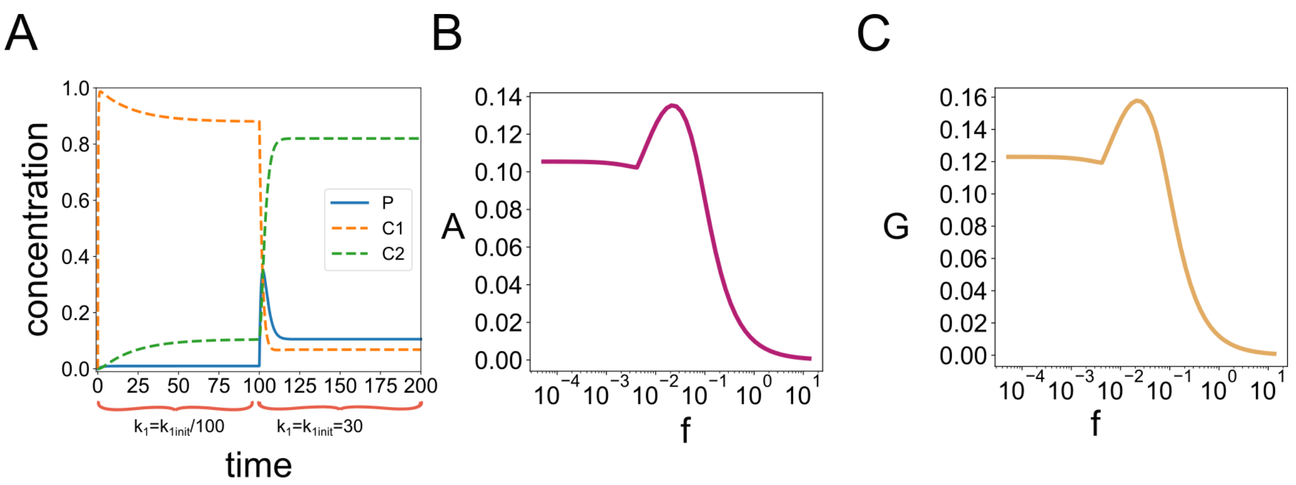

Supplementary Figure 2. Stimulation through k_1. A. Concentrations of P (solid blue line), C_1 (dashed orange line) and C_2 (dashed green line) versus time after a step-like change in S_T and a later step-like change in k_1. Parameter values are: a_1 = 3.5; d_1 = 1; k_{1init} = 30; a_2 = 0.3; d_2 = 0.25; k_2 = 2.5; E_{1T} = 100; E_{2T} = 100; S_T = 35. **B.** Amplitude versus frequency curve, for k_1=30 x (1+sin(ωt))/2. **C.** Gain versus frequency curve.

2. Signal termination and its dependence on the kinetic parameters of the CMC model.

In this section we include the calculations associated to the curves in Fig. 2 in the main text. We first define the Michaelis-Menten constant $K_{M1,2}$:

$$K_{M1,2} = \frac{d_{1,2} + k_{1,2}}{a_{1,2}}$$

where $a_{1,2}$, $d_{1,2}$, $k_{1,2}$ are association, dissociation and catalytic rates, and sub-indexes 1 and 2 correspond to kinase and phosphatase, respectively.

From $K_{M1,2}$, the velocities $V_{1,2}$ and affinities $Aff_{1,2}$ are defined as follows:

$$V_{1,2} = E_{T1,2}\frac{k_{1,2}}{K_{M1,2}} = \frac{a_{1,2}k_{1,2}}{d_{1,2} + k_{1,2}}$$

$$A_{ff\ 1,2} = \frac{1}{K_{M1,2}} = \frac{a_{1,2}}{d_{1,2} + k_{1,2}}$$

We now analytically study the four cases related to Fig. 2.

Case 1:

$a_1, d_1, k_1 = \alpha(a_2, d_2, k_2)$ → $\frac{V_1}{V_2} = \alpha$ and $\frac{A_{ff\,2}}{A_{ff\,1}} = 1$

$a_2, d_2, k_2 = \beta(a_1, d_1, k_1)$ → $\frac{V_1}{V_2} = \frac{1}{\beta}$ and $\frac{A_{ff\,2}}{A_{ff\,1}} = 1$

Case 2:

$a_1 = \alpha a_2$ → $\frac{V_1}{V_2} = \alpha$ and $\frac{A_{ff\,2}}{A_{ff\,1}} = \frac{1}{\alpha}$

$$a_2 = \beta a_1 \quad \rightarrow \quad \frac{V_1}{V_2} = \frac{1}{\beta} \quad \text{and} \quad \frac{A_{ff\,2}}{A_{ff1}} = \beta$$

Case 3:

$$d_1 = \alpha d_2 \quad \rightarrow \quad \frac{V_1}{V_2} = \frac{d_2+k_2}{\alpha d_2+k_2} \quad \text{and} \quad \frac{A_{ff\,2}}{A_{ff1}} = \frac{\alpha d_2+k_2}{d_2+k_2}$$

$$d_2 = \beta d_1 \quad \rightarrow \quad \frac{V_1}{V_2} = \frac{\beta d_1+k_1}{d_1+k_1} \quad \text{and} \quad \frac{A_{ff\,2}}{A_{ff1}} = \frac{d_1+k_1}{\beta d_1+k_1}$$

Case 4:

$$k_1 = \alpha k_2 \quad \rightarrow \quad \frac{V_1}{V_2} = \frac{\alpha(d_2+k_2)}{d_2+\alpha k_2} \quad \text{and} \quad \frac{A_{ff\,2}}{A_{ff1}} = \frac{d_2+\alpha k_2}{d_2+k_2}$$

$$k_2 = \beta k_1 \quad \rightarrow \quad \frac{V_1}{V_2} = \frac{d_1+\beta k_1}{\beta(d_1+k_1)} \quad \text{and} \quad \frac{A_{ff\,2}}{A_{ff1}} = \frac{d_1+k_1}{d_2+\beta k_1}$$

3. Strong signal termination

Strong signal termination conditions are the same as *signal termination* ($P_{max} > 0.1$, $P_{max} > 0.63P_{SS}$) plus $P_{SS} < 0.2$, where P_{max} and P_{SS} are normalized with S_T. In this section we analyze the parameter space exploration in Section 1 in the main text (Fig. 3) but focusing on outputs with strong signal termination and compare them with those corresponding to signal termination. From Supplementary Fig. 2 we conclude that low values of V_1/V_2 and high values of Aff_2/Aff_1 and of E_{2T}/S_T lead to the strong signal termination regime. With E_{1T}/S_T it is not possible to distinguish a region that clearly promotes strong signal termination.

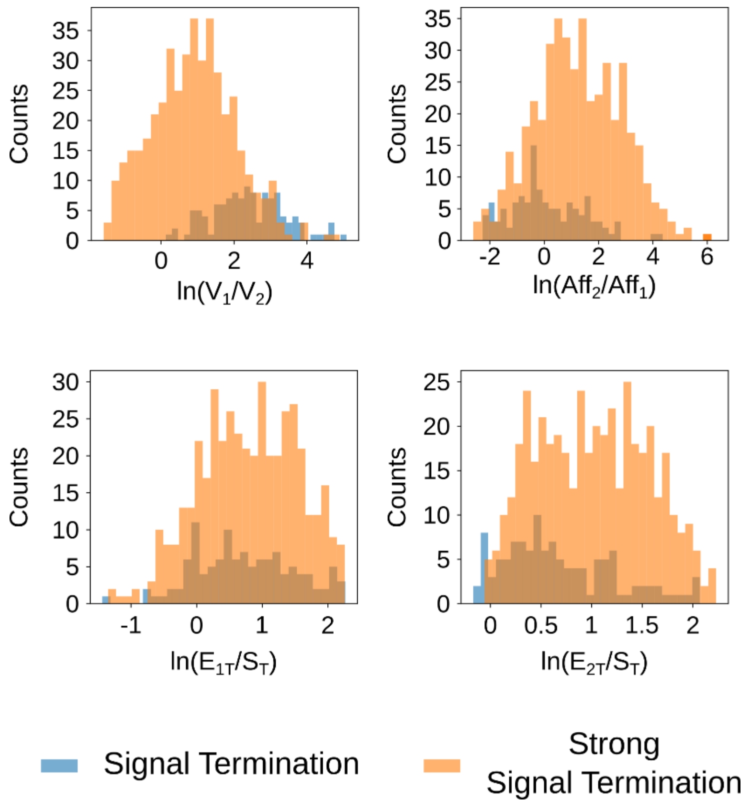

Supplementary Figure 3. Strong signal termination. The outputs of the parameter space exploration in Fig. 3 are plotted as counts versus different parameters combinations. From those outputs we distinguish the group that satisfies the requirement $P_{SS} < 0.2$ and label it as Strong signal termination. The remaining cases are the group exhibiting signal termination with $P_{SS} > 0.2$.

4. Periodic stimulation protocol.

This section is dedicated to better explain different aspects of the periodic stimulation protocol in S_T used in the main text.

As discussed in the main text (Fig. 4), a periodic input in S_T needs to be adapted to avoid negative values in the free form of the substrate. The resulting adapted input signal has a frequency-dependent amplitude. To evaluate if our frequency preference results depend on this amplitude variation, in Supplementary Fig. 4 we expanded Fig. 5 in the main text for different input amplitudes,. The amplitude of the output is sensitive to the amplitude of the input (Supplementary Fig. 4A), while the gain is largely independent (Supplementary Fig. 4B). This is what one would typically expect from a quasi-linear system or a nonlinear system behaving linearly.

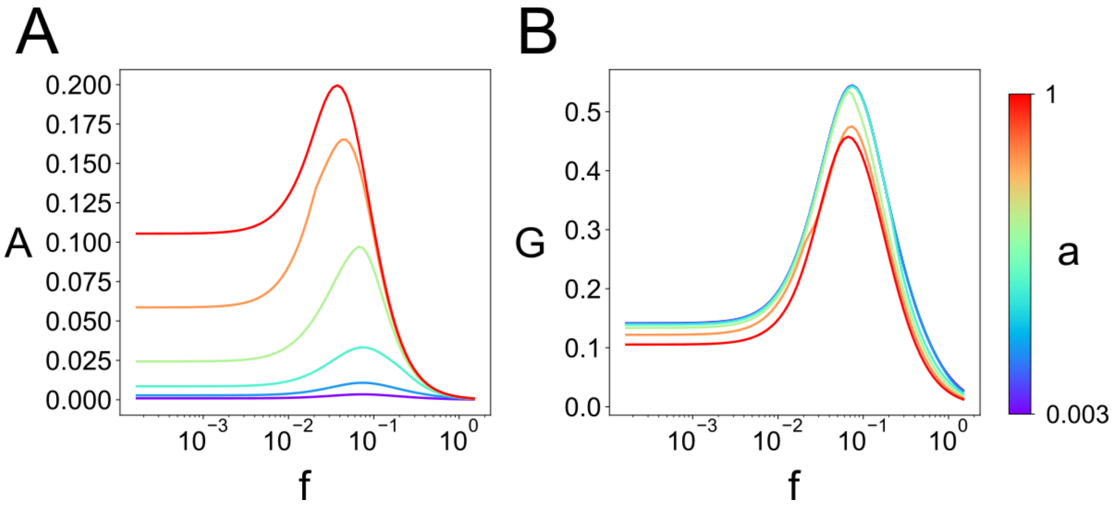

Supplementary Figure 4. Frequency response analysis for different input amplitudes. A and B. Amplitude and gain versus frequency curves for different amplitudes indicated with a color code. The expected input is S_T=35(1+a sin(ωt))/(1+a). Each color curve corresponds to a different value of the amplitude a. Parameter values: a_1 = 3.5; d_1 = 1; k_1 = 30; a_2 = 0.3; d_2 = 0.25; k_2 = 2.5; E_{1T} = 100; E_{2T} = 100; S_T = 35.

Next we study whether the responses measured in terms of the standard impedance (quotient of the Fourier transforms of the output and the input, in absolute value), instead of the metrics we use in the main text., have some type of predictive value. This amounts to compare the impedance profiles (curves of the impedance as a function of the input frequency) for the sinusoidal and square-wave inputs. We show our results in Supplementary Fig. 5. In Supplementary Fig. 5-A1 we show the raw impedances and in Supplementary Fig. 5-A2 we show their filtered versions. While they do not fully coincide as they would for a linear system, they are good approximations of each other in contrast to the peak-to-trough gain shown in the main text (Fig. 5). In Supplementary Figs. 5-B1 and 5-B2 we illustrate that the impedance scales with the input amplitude. This is what one would expect from a linear system and this demonstrates that a study using the impedance would be predictive. Supplementary Fig. 5-C shows other examples of the impedance profiles for other parameter regimes.

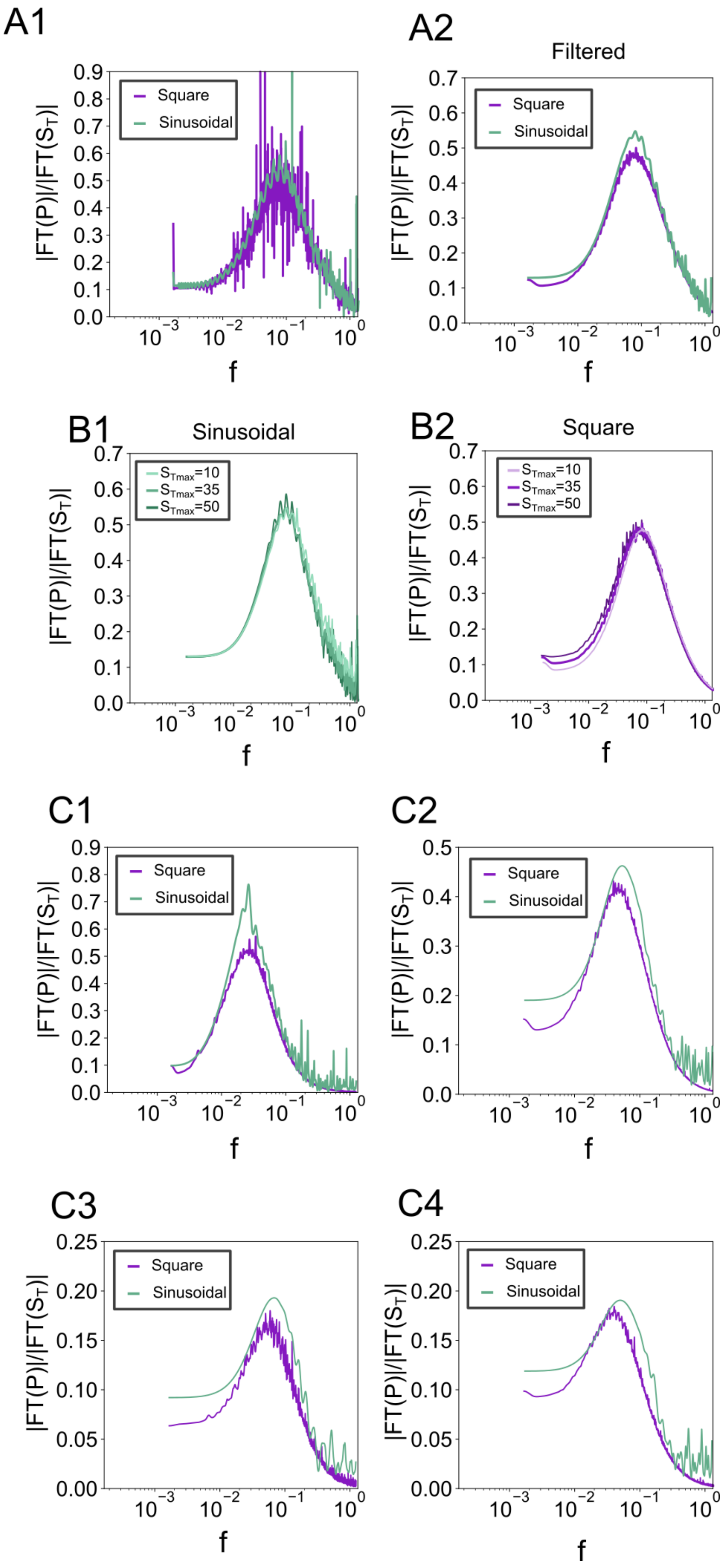
A1
|FT(P)|/|FT(S_T)|
Square
Sinusoidal
f
A2
Filtered
B1
Sinusoidal
S_Tmax=10
S_Tmax=35
S_Tmax=50
B2
Square
C1
C2
C3
C4

Supplementary Figure 5. Impedance versus frequency curves. A1. Raw impedances for sinusoidal (green) and square (purple) input. **A2.** Filtered impedances. **B1-B2.** Filtered impedance for different input amplitudes, for sinusoidal (**B1**) and square (**B2**) inputs. **C.** Impedance profiles for different parameter sets.
Parameter values in A: a_1 = 3.5; d_1 = 1; k_1 = 30; a_2 = 0.3; d_2 = 0.25; k_2 = 2.5; E_{1T} = 100; E_{2T} = 100; S_T = 35. Parameter values in B: S_T=10, 35 and 50.
Parameter values in C: (C1) a_1 = 0.35; d_1 =0.1; k_1 = 20; a_2 = 0.1; d_2 = 0.025; k_2 = 0.5; E_{1T} = 50; E_{2T} = 100; S_T = 35. (C2) a_1 = 2.21; d_1 =0.11; k_1 = 15.4; a_2 = 0.4; d_2 = 0.11; k_2 = 2.3; E_{1T} = 48.7; E_{2T} = 48.6; S_T = 18.3. (C3) a_1 = 0.76; d_1 =0.61; k_1 = 36.5; a_2 = 1.33; d_2 = 0.1; k_2 = 3.9; E_{1T} = 21.5; E_{2T} = 43.7; S_T = 17.5. (C4) a_1 = 4.46; d_1 =2.32; k_1 = 7.47; a_2 = 0.44; d_2 = 0.95; k_2 = 2.64; E_{1T} = 19.2; E_{2T} = 66.5; S_T = 14.8.
Except for A1, all the panels are plotted with a Gaussian smoothing filter with smoothing parameter *s*=80 for sinusoidal input, and *s*=10 for square input. FT was calculated with function fft from library fftpack and the Gaussian filter was calculated with function gaussian_filter from package ndimage, both from Spicy, Python.

We describe here an alternative way of adapting the expected S_T signal. We produce an adapted input that not only has the same period as the expected one, but it also has its maxima and minima at the same time as the expected input. The adapted signal used in the main text shares the locations of the maxima with the expected input, but not the locations of the minima. In Supplementary Fig. 6A we compare these two ways of adapting the input signal (so to avoid negative values in the systems' variables). In Supplementary Figs. 6B and C we plot the amplitude and gain versus frequency for both types of adapted signals, and in Supplementary Fig. 6D we compare the distance between expected and adapted signals. The alternative way of adapting the expected signal produces preferred frequency responses in the gain, but not in the amplitude.

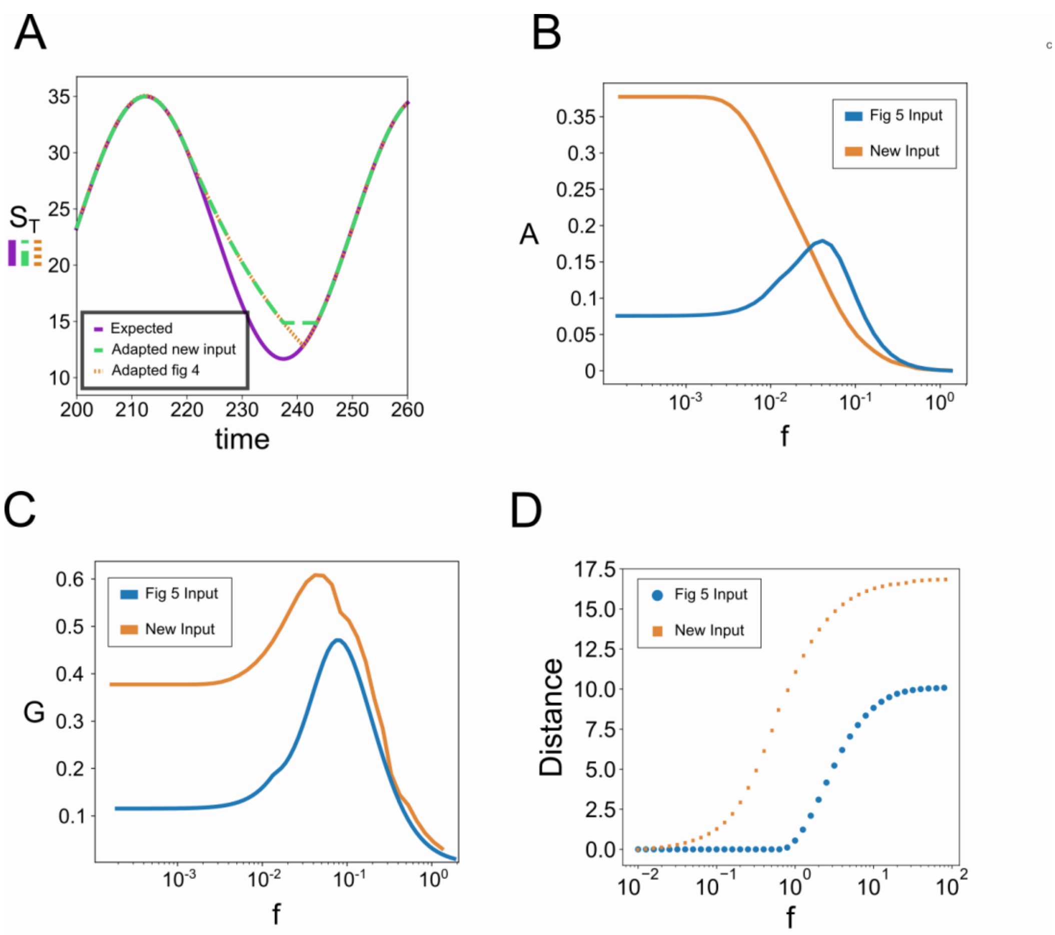

Supplementary Figure 6. Alternative adapted stimulation. A. Adapted (dashed green line for the new adapted input, solid orange line for the previous adapted input) and expected (solid purple line) input signals versus time. Solid yellow line indicates free *S*. The input has *T* = 50s. **B and C.** Frequency response curves, measuring amplitude (B) and gain (C) as a function of the input frequency. Orange for the new adapted input, blue for the one included in the main text. **D.** Distance between the expected input and each of the adapted ones, as a function of the input frequency. Distance is calculated as $D = \frac{1}{T}\int_{t^*}^{t^*+T}\left(S_{TExpected} - S_{TAdapted}\right)dt$ where t^* is a time for which the system has reached the periodic regime. Parameter values: a_1 = 3.5; d_1 = 1; k_1 = 30; a_2 = 0.3; d_2 = 0.25; k_2 = 2.5; E_{1T} = 100; E_{2T} = 100; S_T = 35.

Finally, we include an analytical calculation that clarifies the ideas behind the need of adapting the forcing S_T (from expected to adapted) due to the sequestration of some components appearing in the conservation law. This calculation is done in a ligand-receptor reaction, an scenario that is simpler than a CMC, where analytical calculations are not possible.

Periodic stimulation in a ligand-receptor reaction

We consider a ligand L that binds a receptor R forming a complex C, with binding and unbinding rates k_{on} and k_{off}, respectively. This reaction is described by the following equation:

$$\frac{dC}{dt} = k_{on}LR - k_{off}C$$

and the two conservation laws:

$L_T = L + C, \quad R_T = R + C.$

Let us consider that the ligand is in excess, so that $L \sim L_T$, and that there is a periodic forcing in R_T. The differential equation describing the reaction results is given by

$$\frac{dC}{dt} = k_{on} L_T (R_T - C) - k_{off} C$$

This can be expressed as:

$$\frac{dC}{dt} = \alpha R_T - \beta C$$

where α and β are constants. For the input $R_T(t) = R_0(1 + a sin(\omega t))$, the particular solution for C is:

$$C_P(t) = \frac{\alpha R_0}{\beta} + \frac{\beta a \alpha R_0}{\omega^2 + \beta^2} sin(\omega t) - \frac{\alpha R_0 a \omega}{\omega^2 + \beta^2} \cos(\omega t)$$

Using the conservation law for R_T, we obtain the following expression for (free) R:

$$R(t)/R_0 = \left(1 + sin(\omega t)\right) - \frac{\alpha}{\beta} - \frac{\beta \alpha a}{\omega^2 + \beta^2} sin(\omega t) + \frac{\alpha a \omega}{\omega^2 + \beta^2} \cos(\omega t)$$

We then analyze if this expression is always positive. We consider a time such that $t^* = \frac{\pi}{2\omega}$, resulting in $\cos(\omega t^*) = 0$ and $\sin(\omega t^*) = -1$:

$$R/R_0 = \left(1 - \frac{\alpha}{\beta}\right) + a\left(\frac{\beta \alpha}{\omega^2 + \beta^2} - 1\right)$$

We observe that it cannot be guaranteed that this expression is always positive, for every parameter choice. Particularly, it becomes negative for every frequency satisfying the following condition:

$$\frac{\left(\beta \alpha \left(1 - \frac{1}{a}\right) - \beta^2 \left(1 - \frac{1}{a}\right)\right)}{\left(\frac{\alpha}{\beta a} - \frac{1}{a} + 1\right)} < \omega^2$$

For example, for α=1, β=1.1 and a=0.5, it turns negative for ω=0.37, and for α=1.2, β=1.5 and a=0.9, it turns negative for ω=0.25.

The ligand-receptor model analyzed above corresponds to a (ligand-receptor) reaction in which the ligand is in excess and where a periodic forcing is applied in R_T (total amount of receptor), which is a conserved quantity. This simple model is analogous to, but simpler than the model we use in the article, where the enzymes are in excess so the periodic forcing is applied in the limiting specie, S_T (total amount of substrate). By carrying out simple calculations we showed that a conserved quantity that is mostly sequestered, because the other interacting species are in excess, could generally not follow a desired profile with a desired kinetics. The ligand-receptor model is not only useful to provide relevant intuition, but it is also exactly equal to that one arising from a particular limiting scenario in CMCs, the so called "weakly activated" form, as described by Heinrich et al (1). In this sense, the ligand-receptor model is a limiting case of the CMC model we use in this paper.

5. Robustness of the results for different parameter values and parameter ranges.

As explained in the main text, some of the studies are performed over specific, representative parameter sets (Figs. 1, 5, and 7), while others are based on a parameter space exploration (Figs. 3, 6, and 8). The representative parameter sets capture scenarios that have a more general validity. For this first group, we here include the results of alternative parameter sets, showing that the conclusions extracted from those studies are independent from the choice of parameter set (Supplementary Fig. 7).

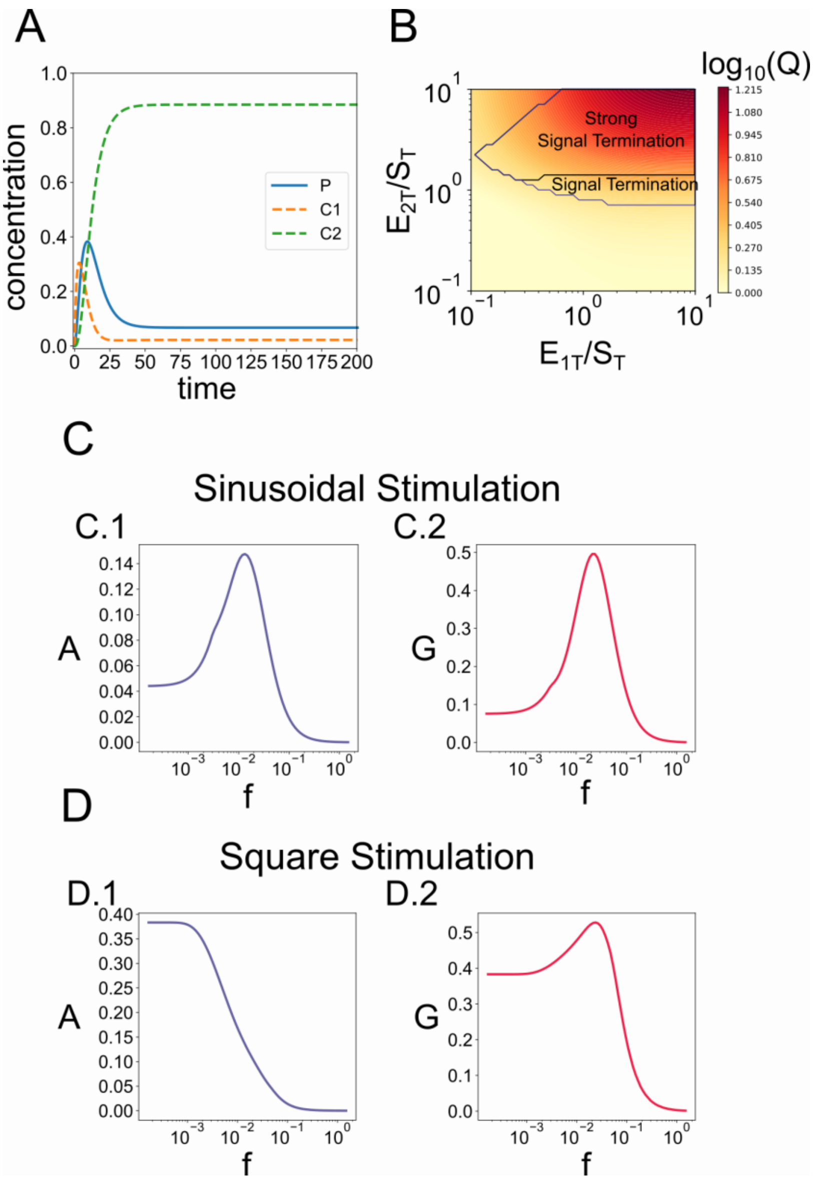

Supplementary Figure 7. Results with alternative parameter sets. Parameter values: a_1=0.35, d_1=0.1, k_1=20, a_2=0.1, d_2=0.025, k_2=0.5, E_{1T}=50, E_{2T}=100, S_T=35. These values result in V_1=0.35, V_2=0.1; Aff_1=0.02, Aff_2=0.19. **A.** P, C_1, C_2 temporal profiles for step-like stimulation. **B.** Graph of Q (= P_{max}/P_{ss}) versus E_{1T}/S_T and E_{2T}/S_T, with Q in colorscale. The solid blue line over the plots corresponds to Q=1.6 and separates signal termination from monotonic behavior and non-monotonic behavior not satisfying the criteria. The solid black line over the plot corresponds to P_{ss} = 0.2. The cases over the line (P_{ss} > 0.2) are those termed strong signal termination. **C and D.** Frequency response results, measuring amplitude (left) and gain (right) as a function of the input frequency. C corresponds to adapted sinusoidal stimulation, D corresponds to adapted train of square pulses stimulation.

In Supplementary Fig. 8 we evaluate the impact of the parameter ranges used for the random parameter space exploration. The study in the main text is done using the following ranges: E_{1T}, E_{2T}, and S_T in 10-100, the association and dissociation rates (a_1, d_1, a_2, d_2) in 0.1-10 and the catalytic rates (k_1, k_2) in 1-50. We here modify the ranges for the catalytic rates, making them equal to the other rates, i.e., 0.1-10. The results are in Supplementary Fig. 8 and lead to the same conclusions extracted from Fig. 3 in the main text.

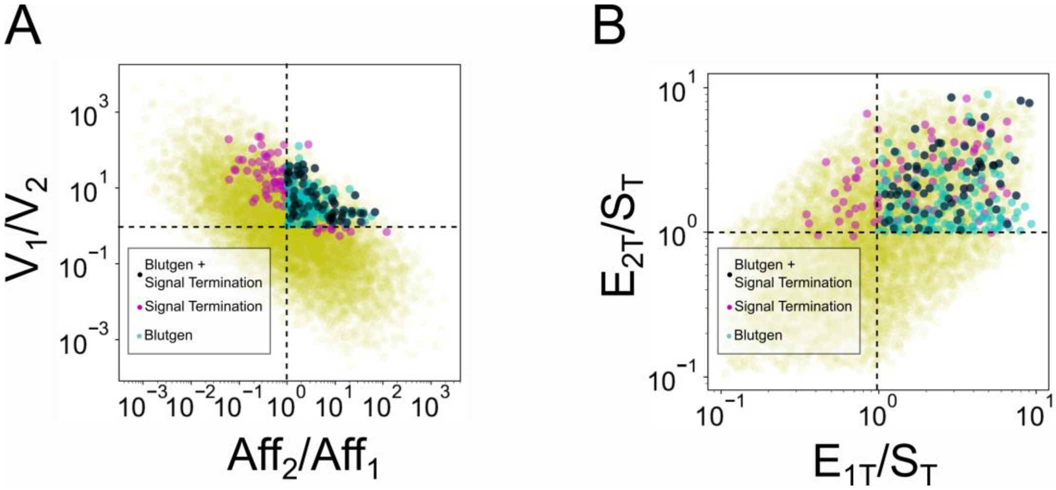

Supplementary Figure 8. Parameter space exploration with different ranges for the catalytic rates. A. V_1/V_2 - Aff_2/Aff_1 parameter space, **B**. E_{2T}/S_T - E_{1T}/S_T parameter space. Both panels include the output of numerical simulations for 10,000 parameter sets in the selected ranges of variation. Each dot represents a single simulation output. Dots in purple (N=147) correspond to outputs with signal termination, dots in cyan (N=431) correspond to the parameter sets that satisfy Bluthgen's conditions, dots in black (N=60) correspond to the parameter sets that satisfy Bluthgen's conditions and signal termination. All the other simulation results are in yellow. Outputs lower than 0.1 were excluded from the analysis. Parameter ranges: E_{1T}, E_{2T}, and S_T in 10-100, association, dissociation and catalytic rates (a_1, d_1, a_2, d_2, k_1, k_2) in 0.1-10.

Most of the studies in the paper are done with the transition from S_T=0 to a higher value of S_T. We include here a study of the transition between two non-zero S_T values. As can be seen in Supplementary Fig. 9A, signal termination is still obtained. Supplementary Figs. 9B and C exhibit the parameter space exploration results, which lead to the same conclusions as those in Figs. 1 and 3 in the main text.

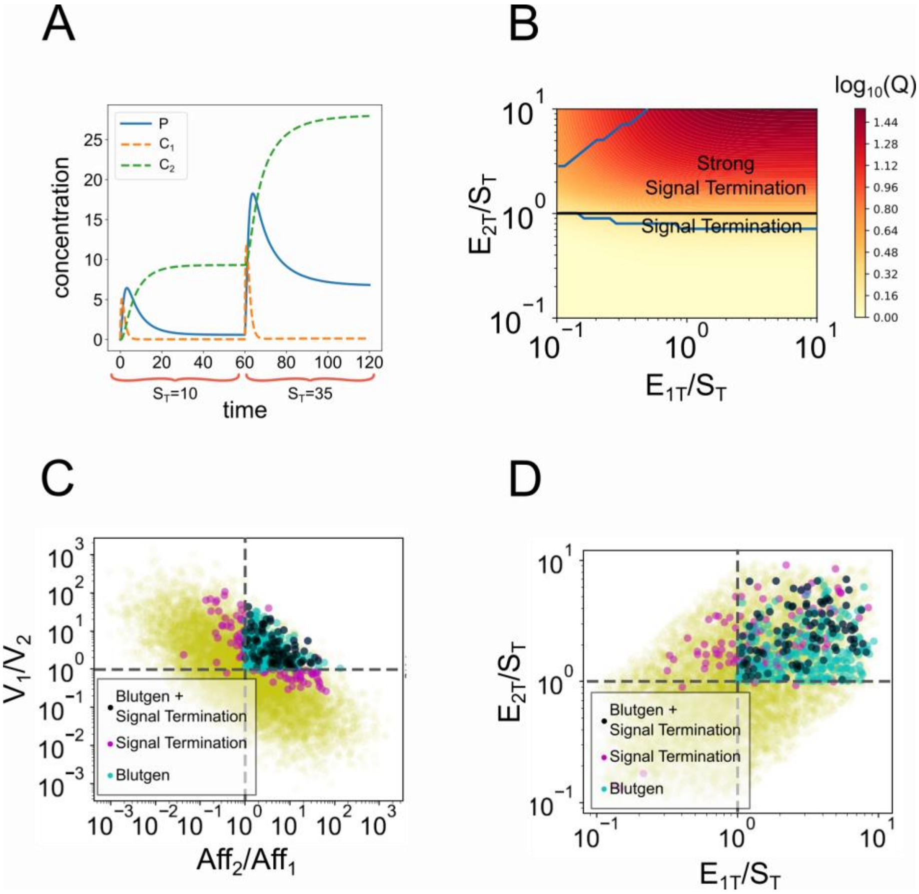

Supplementary Figure 9. Step-like stimulation from a non-zero value. A. P, C_1, C_2 temporal profiles for step-like stimulation. **B.** Graph of Q versus E_{1T}/S_T and E_{2T}/S_T, with Q in colorscale. The solid blue line over the plot separates signal termination from monotonic behavior and non-monotonic behavior not satisfying the criteria. The solid black line over the plot corresponds to P_{ss} = 0.2. **C.** V_1/V_2 - Aff_2/Aff_1 parameter space. **D**. E_{2T}/S_T - E_{1T}/S_T parameter space. Both panels include the output of numerical simulations for 10,000 parameter sets in the selected ranges of variation. Each dot represents a single simulation output. Dots in purple (N=200) correspond to outputs with signal termination, dots in cyan (N=448) correspond to the parameter sets that satisfy Bluthgen's conditions, dots in black (N=97) correspond to the parameter sets that satisfy Bluthgen's conditions and signal termination. All the other simulation results are in yellow. Outputs lower than 0.1 were excluded from the analysis. Parameters in A and B are as in Fig. 1 in the main text, stimulation goes from 0 to S_T=10 for t < 60s, and from S_T=10 to S_T=35 for t > 60s. Parameter ranges in C and D are as in Fig. 3 in the main text, for each set where S_T is randomly chosen, the step in S_T goes from S_T/3.5 to S_T.

6. Cascades of covalent modification cycles

In this section we include two additional figures related to the study of cascades of CMCs. In Supplementary Fig. 10 we plot the temporal profiles of the different variables involved in both an isolated and a coupled CMC, to better understand the behaviors described in Fig. 8 in the main text. We can see that the sequestration of the product of the first cycle in complex C_2 produces signal termination in the coupled system. In Supplementary Fig. 11 we include details of the frequency preference response in cascades of CMCs.

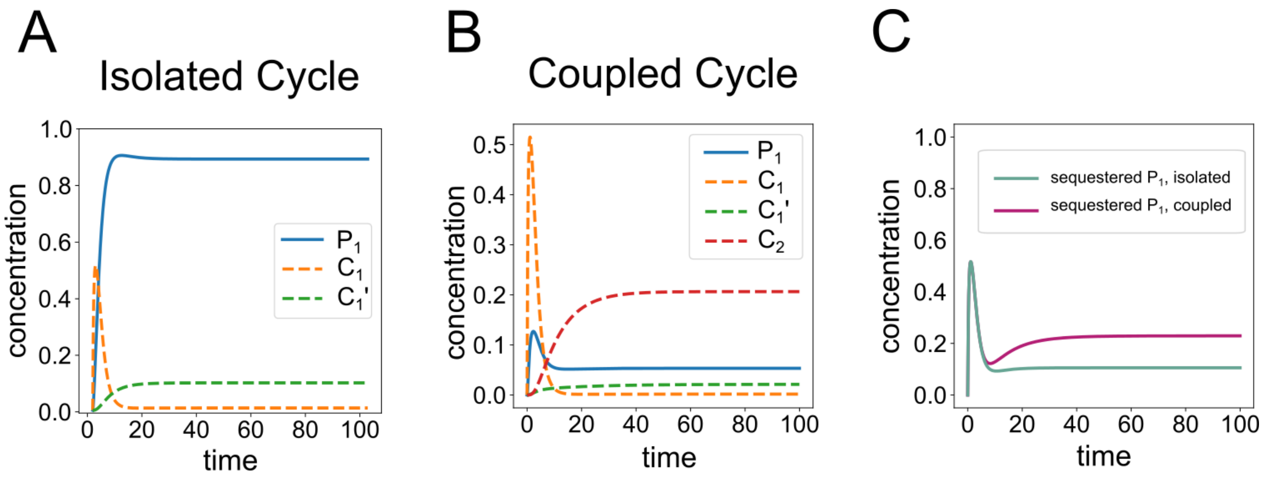

Supplementary Figure 10. Signal termination induced by sequestration in cascades of CMCs. A. and B. Temporal profiles for the isolated (A) and coupled (B) CMC. P_1 in solid blue lines, C_1 in dashed orange lines, C_1' in dashed green lines, C_2 in dashed red lines. Parameter values in A: a_1 = 3.5; d_1 = 1; k_1 = 30; a_1' = 0.3; d_1' = 0.25; k_1' = 2.5; E_{1T} = 35; E_{1T}' = 4.4; S_{1T} = 35. Parameter values in B: a_1 = 3.5; d_1 = 1; k_1 = 30; a_1' = 0.3; d_1' = 0.25; k_1' = 2.5; E_{1T} = 35; E_{1T}' = 4.4; S_{1T} = 35; a_2=1.56; d_2=2.34; k_2=1.52; a_2'=1.87; d_2'=1.42; k_2'=6.80; E_{2T}'=73.77; S_{2T}=83.02. **C** Sequestered P_1 for isolated (green) and coupled (purple) CMCs.

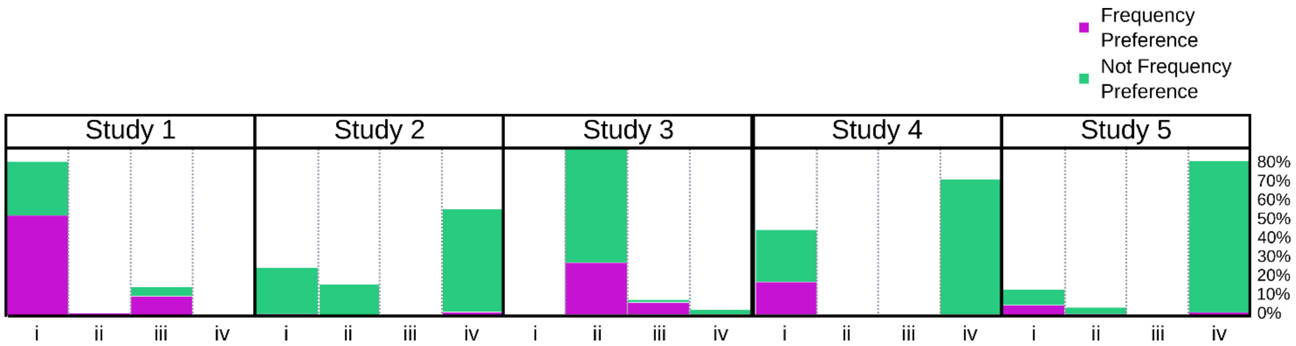

Supplementary Figure 11. Frequency preference response in cascades of CMCs. Summary of frequency preference responses in the five studies described in the main text, Fig. 8. Each bar is now colored according to the fraction of outputs resulting in a frequency preference response (purple) or not (green). For columns i, the frequency preference response is evaluated in the first cycle; for columns ii, iii, and iv, the frequency preference response is evaluated in the second cycle.

7. Mathematical approximations for CMCs models

CMC dynamics described by using a quasi-steady-state approximation (QSSA). The quasi-steady state approximation is frequently used to derive reduced models for enzyme-catalyzed reaction networks. The QSSA relies on the assumption that the enzyme-substrate complexes rapidly approach a quasi-steady state, leading to an algebraic relation for those complexes. Under the additional assumption that the substrate is in excess over the enzymes, one can derive the following ODE for P, the modified substrate (Goldbeter & Koshland, 1981):

$$\frac{dP}{dt} = k_1 E_{1T} \frac{(S_T - P)}{K_1 + (S_T - P)} - k_2 E_{2T} \frac{P}{K_2 + P}$$

where $K_{1,2} = \frac{d_{1,2}+k_{1,2}}{a_{1,2}}$.

The applicability of this reduced model is restricted to conditions when the substrate concentration is much higher than that of the converter enzymes. However, while this procedure mostly preserves the steady-state structure of the network, by their own assumptions, it often fails to correctly capture its transient dynamics.

CMC dynamics described with a total quasi-steady-state approximation (tQSSA). This approximation is based on certain linear combinations of the original variables and has proven to yield much better approximations than the standard QSSA, especially when the enzyme concentration becomes comparable to or larger than that of the substrate (Straube, 2017). For a single CMC, the tQSSA equations are given by (Straube, 2017) (see Eqs. (18), (21) and (22) there)

$$\frac{dP}{dt} = k_1 C_1 - k_2 C_2$$

$$C_1 = \frac{E_{1T} + S_T - P + K_1}{2} + \sqrt{\frac{(E_{1T} + S_T - P + K_1)^2}{4} - E_{1T}(S_T - P)}$$

$$C_2 = \frac{E_{2T} + P + K_2}{2} + \sqrt{\frac{(E_{2T} + P + K_2)^2}{4} - E_{2T}P}$$

8. Parameter values used in the figures

Parameter	Units	Fig 1 Signal Termination	Fig 4 Fast Kinetics	Fig 4 Slow Kinetics	Fig 5/Fig 9 Frequency Preference/Fig 7 /Fig 10 Approximations
a_1	1/(conc × min)	3.50	3.50	0.35	3.50
d_1	1/min	1	1	1	1
k_1	1/min	50	30	30	30
a_2	1/(conc × min)	0.30	0.30	0.03	0.30
d_2	1/min	0.25	0.25	0.25	0.25
k_2	1/min	0.25	2.50	2.50	2.50
E_{1T}	conc	100	100	100	100
E_{2T}	conc	100	100	100	100
S_T	conc	35	35	35	35

Fig. 8

Parameter	Units	Study 1	Study 2	Study 3	Study 4	Study 5
a_1	1/(conc × min)	3.50	3.50	random	random	random

d_1	1/min	1	1	random	random	random
k_1	1/min	30	30	random	random	random
a_1'	1/(conc × min)	0.30	0.30	random	random	random
d_1'	1/min	0.25	0.25	random	random	random
k_1'	1/min	2.50	2.50	random	random	random
a_2	1/(conc × min)	random	random	3.50	3.50	random
d_2	1/min	random	random	1	1	random
k_2	1/min	random	random	30	30	random
a_2'	1/(conc × min)	random	random	0.30	0.30	random
d_2'	1/min	random	random	0.25	0.25	random
k_2'	1/min	random	random	2.50	2.50	random
E_{1T}	conc	100	35	random	random	random
E_{1T}'	conc	100	4.40	random	random	random
E_{2T}'	conc	random	random	100	1	random
S_{1T}	conc	35	35	random	random	random
S_{2T}	conc	random	random	35	35	random

Fig 9

**Parameter**	**Units**	**C1**	**C2**	**C3**	**C4**
a_1	1/(conc × min)	3.50	3.50	0.5	3.50
d_1	1/min	1	1	1.08	1
k_1	1/min	30	30	1.82	30
a_1'	1/(conc × min)	0.30	0.30	0.12	0.30
d_1'	1/min	0.25	0.25	2.93	0.25
k_1'	1/min	2.50	2.50	18.58	2.50
a_2	1/(conc × min)	2.73	0.23	3.5	1.32
d_2	1/min	0.51	0.30	1	1.55
k_2	1/min	33.84	12.90	30	49.88
a_2'	1/(conc × min)	0.20	0.68	0.3	2.61
d_2'	1/min	0.17	2.25	0.25	4.10
k_2'	1/min	1.19	19.18	2.5	16.29
E_{1T}	conc	100	100	41.23	100
E_{1T}'	conc	100	100	72.8	100
E_{2T}'	conc	76.84	12.28	100	17.07
S_{1T}	conc	35	35	13.59	35
S_{2T}	conc	53.51	28.07	35	13.21

Supplementary References

1. Heinrich R, Neel BG, Rapoport T a. Mathematical models of protein kinase signal transduction. Mol Cell. 2002;9(5):957–70. Available from: https://doi.org/10.1016/S1097-2765(02)00528-2
